# Copy number variation in tRNA isodecoder genes impairs mammalian development and balanced translation

**DOI:** 10.1038/s41467-023-37843-9

**Published:** 2023-04-18

**Authors:** Laetitia A. Hughes, Danielle L. Rudler, Stefan J. Siira, Tim McCubbin, Samuel A. Raven, Jasmin M. Browne, Judith A. Ermer, Jeanette Rientjes, Jennifer Rodger, Esteban Marcellin, Oliver Rackham, Aleksandra Filipovska

**Affiliations:** 1 Harry Perkins Institute of Medical Research, Perth, WA Australia; 2 ARC Centre of Excellence in Synthetic Biology, QEII Medical Centre, Nedlands, WA 6009 Australia; 3 Centre for Medical Research, The University of Western Australia, QEII Medical Centre, Nedlands, WA 6009 Australia; 4 Australian Institute for Bioengineering and Nanotechnology, The University of Queensland, Brisbane, 4072 QLD Australia; 5 Monash Genome Modification Platform, Monash University, 35 Rainforest Walk, Clayton, VIC 3800 Australia; 6 School of Biological Sciences (Physiology), The University of Western Australia, Crawley, WA 6009 Australia; 7 Perron Institute for Neurological and Translational Sciences, Nedlands, WA 6009 Australia; 8 Queensland Metabolomics and Proteomics (Q-MAP), The University of Queensland, Brisbane, 4072 QLD Australia; 9 Curtin Medical School, Curtin University, Bentley, WA 6102 Australia; 10 Curtin Health Innovation Research Institute, Curtin University, Bentley, WA 6102 Australia; 11 Telethon Kids Institute, Northern Entrance, Perth Children’s Hospital, 15 Hospital Avenue, Nedlands, WA Australia

**Keywords:** tRNAs, RNA, Development, Embryology

## Abstract

The number of tRNA isodecoders has increased dramatically in mammals, but the specific molecular and physiological reasons for this expansion remain elusive. To address this fundamental question we used CRISPR editing to knockout the seven-membered phenylalanine tRNA gene family in mice, both individually and combinatorially. Using ATAC-Seq, RNA-seq, ribo-profiling and proteomics we observed distinct molecular consequences of single tRNA deletions. We show that *tRNA-Phe-1-1* is required for neuronal function and its loss is partially compensated by increased expression of other tRNAs but results in mistranslation. In contrast, the other *tRNA-Phe* isodecoder genes buffer the loss of each of the remaining six *tRNA-Phe* genes. In the *tRNA-Phe* gene family, the expression of at least six *tRNA-Phe* alleles is required for embryonic viability and *tRNA-Phe-1-1* is most important for development and survival. Our results reveal that the multi-copy configuration of tRNA genes is required to buffer translation and viability in mammals.

## Abstract (web summary)

Enigmatically tRNA genes exist in several hundred copies in mammalian genomes. Here the authors find a precipitous failure of development and increased amino acid misincorporation upon systematic elimination of tRNA-Phe genes in mice using CRISPR.

## Introduction

**Fig. 1:**
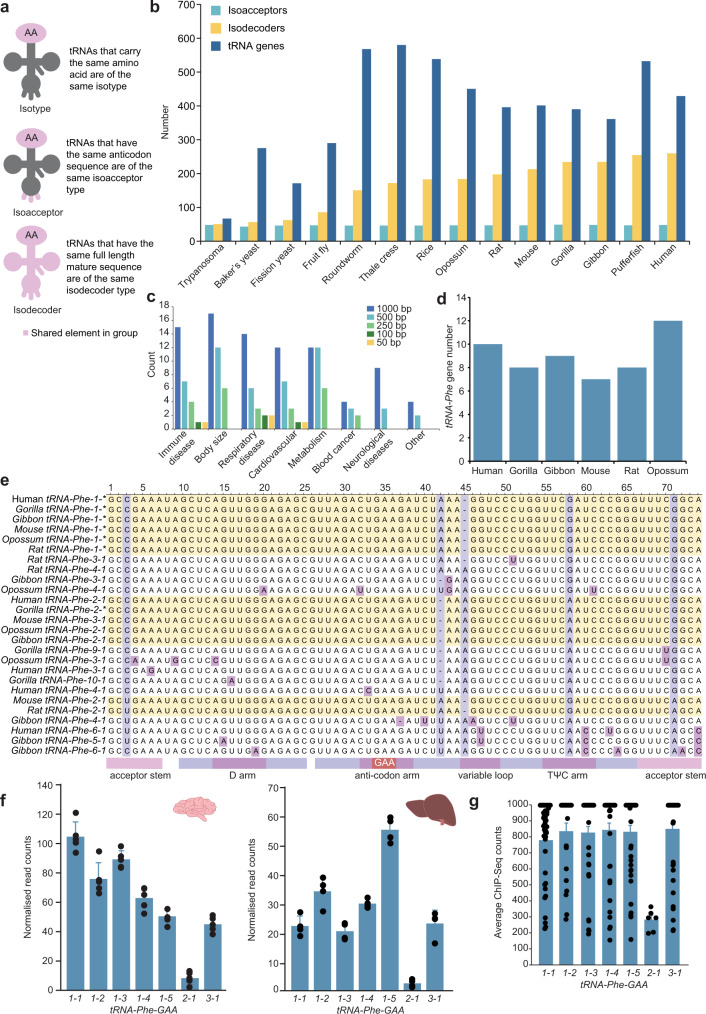
Evolutionary diversity and distribution of mammalian multigene tRNA families. **a** Classes of tRNA familes. A tRNA isotype refers to a tRNA that is charged with one of the same 20 common amino acids, isoacceptor tRNAs are tRNAs that have distinct anticodons but are charged with the same amino acid, and isodecoders have the same anticodon but sequence differences in the rest of the tRNA body. **b** Number of tRNA isoacceptor and tRNA isodecoders in diverse species as listed by GtRNAdb. **c** GWAS associations in close proximity to human *tRNA-Phe-GAA* genes with number of significant GWAS hits (*p* < 0.05, one-way ANOVA) within up- and down-stream windows of ±50, ±100, ±250, ±500 and ±1000 bp of the tRNA gene. Individual disease annotations were summarised into disease categories. **d** Numbers of *tRNA-Phe* genes identified across six species of mammals from GtRNAdb. **e** Alignment of *tRNA-Phe* gene sequences from six mammalian species. Identical sequences within a species were collapsed and indicated by*. For example, mouse *tRNA-Phe-1-1*, *1-2*, *1-3*, *1-4* and *1-5* all have identical sequences and have been collapsed, represented in the figure as Mouse tRNA-Phe-1-*. Horizontal yellow bars highlight identical sequence clusters, vertical purple bars show variability at that position and pink squares indicate single or low represented sequence variations. Secondary tRNA structural information is annotated at the bottom of the alignment. **f** Chromatin accessability of the seven *tRNA-Phe* genes in brain and liver samples isolated from 5 control mice in brain and 4 control mice in liver, determined by ATAC-Seq. All values are means ± SD. **g** Occupancy of RNA polymerase III at the promoters of the seven *tRNA-Phe* genes determined by ChIP-Seq (ChIP-Atlas). *tRNA-Phe-1-1* *n* = 52, *1-2* *n* = 24, *1-3* *n* = 54, *1-4* *n* = 40, *1-5* *n* = 40, *2-1* *n* = 6 and *3-1* *n* = 51. Values are means ± SD.

Transfer RNAs (tRNAs) are the most abundant RNAs and define the genetic code by setting the rules for translation of RNA into protein. tRNAs are relics from the RNA world^1^, suggested to have originated from one of the most ancient RNAs and subsequently adopted to transfer amino acids^2^. tRNAs carry amino acids at their 3′ end and decode mRNAs by complementing each codon within messenger RNAs (mRNAs) to their anticodons to position amino acids for polymerisation on ribosomes. tRNA isotypes are loaded with one of the 20 amino acids and can be classified as different isoacceptors, tRNAs that use synonymous codons for the same amino acid, and different classes of isodecoders (Fig. 1a), tRNAs that use the same codon but differ in the tRNA body, each of which may be transcribed from one or more tRNA genes. The importance of tRNA isoacceptors was appreciated even before the first genome was sequenced, as tRNAs recognising the same amino acid were found as a population of molecules with different sequences by direct sequencing^3,4^. Since then a comprehensive body of evidence has accumulated that demonstrates the importance of the abundance of different isoacceptors in dictating the efficiency and fidelity of translation for mRNAs with different codon usage^5,6^. However, the preponderance of tRNA isodecoders – making up more than half of all tRNA genes observed in higher mammals – was unexpected and its functional importance is not yet understood^6,7^.

Bioinformatic mining of genome sequences for tRNA genes revealed that the number of tRNA isodecoders varies dramatically, ranging between 3.6 and 55% of tRNAs in different organisms, with very few observed in microbes, an expansion in multicellular organisms, and the highest numbers observed in mammals^7^. Currently, there is no mechanistic explanation for the increased frequency of isodecoder tRNAs or the genes that encode them. Hypotheses include neutral drift concomitant with genome expansion^7^, that tRNA genes spatially separate different genomic domains and provide fragile sites to accelerate genome evolution^8^, or that different tRNAs are expressed in a tissue-specific manner^9^ or have acquired distinct functions in translation^10^. However, there is little experimental evidence to support these hypotheses beyond correlative studies^11^. Nevertheless, different evolutionary pressures have given rise to diverse tRNA genes and in vitro studies have shown that differences in the sequences of the tRNA body can modulate the aminoacylation efficiency and fidelity of protein synthesis^12,13^, which may be amenable for specific processes such as facilitating protein folding or biogenesis of large protein complexes^14,15^. In yeast, it has been shown that loss of an essential or unique isoacceptor can be compensated by a single-nucleotide mutation in the anticodon in an isodecoder tRNA^16^, indicating that the multi-gene families of tRNAs could be used to meet different translational demands under specific evolutionary or environmental pressures. Therefore, the large pool of tRNA genes may provide an evolutionary advantage that could be important for the fidelity of translation.

To address the fundamental questions regarding the role of multi-gene isodecoder tRNA families and specific tRNA requirements in mammals we used CRISPR-Cas9 to delete every member of the phenylalanine tRNA gene family in mice. Detailed molecular and phenomic characterisation, either as single knockouts or multiple gene knockouts, determined the importance of gene copy number and the specific isodecoder gene requirements for survival and fine-tuning of organismal function.

## Results

### Diversity of mammalian *tRNA-Phe* isodecoder genes

There is large disparity in tRNA gene distribution in eukaryotes; with a similar number of isoacceptors (up to 55) compared to a varying number of isodecoders ranging from one in trypanosomes to hundreds in vertebrates (Fig. 1b). In mammals, tRNA genes are typically distributed in clusters and particularly enriched on specific chromosomes (Supplementary Fig. 1a). For example, the majority of human tRNA genes are located on chromosome 6, while chromosome 13 contains the highest number of tRNA genes in mice (Supplementary Fig. 1a). Since mutations in tRNA genes can contribute to disease^17^, we analysed public GWAS datasets to find that variations within close proximity to human tRNAs were implicated in body size variation, as well as immune disease, respiratory diseases and metabolism (Fig. 1c), implicating tRNA variations as genetic contributors to normal variation and also potentially disease.

The total tRNA gene number in different species of mammals ranges from 361 in gibbons to 450 in opossums^7^ (Supplementary Fig. 1b). Isoacceptor numbers remain consistent across the species we analysed, while isodecoder numbers range from the lowest in opossums (182) to the highest number in humans (258). The tRNA gene family for incorporation of phenylalanine (*tRNA*^*Phe*^*(GAA)*, henceforth *tRNA-Phe*) has the fewest genes in mammals (Fig. 1d), simplifying genetic editing. In mice there are 7 tRNA genes that make up the *tRNA-Phe* family, that all have the same GAA anticodon. These tRNA genes fall into three isodecoder subfamilies, *tRNA-Phe-1*, that comprises five genes, *tRNA-Phe-2* and *tRNA-Phe-3*, each represented by one gene in mice. Although there are elevated rates of mutation in tRNA flanking regions, mature tRNA gene sequences are highly conserved^18^, and we found that mature *tRNA-Phe* genes for members of the isodecoder tRNA family 1 (*tRNA-Phe-1*) had 100% sequence similarity (Fig. 1e). Mouse and rat *tRNA-Phe-2-1* were also identical (Fig. 1e). Interestingly, human, gorilla, opossum and gibbon *tRNA-Phe-2-1* and mouse *tRNA-Phe-3-1* had conserved sequences, suggesting that this tRNA may perform similar functions within these species compared to the other mouse *tRNA-Phe* genes (Fig. 1e). All the remaining *tRNA-Phe* genes were unique, with the opossum *tRNA-Phe-3-1* containing the highest number of single-base variations, having four distinct variants across the gene sequence. Variations are present in all secondary structure regions, however, a single region spanning 11 bp between the end of the D loop and the start of the anticodon loop is the longest completely conserved sequence between all species. This particular section may be important for tRNA function, due to the position of the D-arm on the interior angle of the tRNA tertiary structure, between the anticodon and acceptor stem, and may have been preserved via purifying selection. Pairwise analyses of syntenic regions showed the divergence of specific *tRNAPhe* genes across the six mammalian species (Supplementary Fig. 1c).

tRNA expression is highly dependent on chromatin state and relies on promoters internal to the tRNAs themselves, therefore, we used assay for transposase-accessible chromatin sequencing (ATAC-Seq) to measure chromatin accessibility as a gene-specific proxy for the expression levels of *tRNA-Phe* genes in the brain and liver of wild-type mice (Fig. 1f and Supplementary Fig. 1d, e), as it has been done previously^19^. Both hybridisation-based techniques and RNA sequencing can provide total quantitation for specific tRNAs with the same anticodon but none of these methods are able to discriminate between specific anticodon groups and isodecoders that differ by one or more bases. RNA sequencing also encounters the same problem with assigning the identical or near identical sequences to different loci, which is the reason we carried out ATAC-Seq to accurately assign specific tRNA-Phe genes to their loci in the genome. We found notable variation in the accessibility of the *tRNA-Phe* genes, where *tRNA-Phe-1-1* and *tRNA-Phe-1-2* had the highest signal and *tRNA-Phe-2-1* had the lowest signal in both tissues (Fig. 1f). Our ATAC-Seq findings were further validated by publicly available liver ChIP-seq data^20^ (Fig. 1g).

### CRISPR-Cas9 editing of single *tRNA-Phe* family genes results in tissue-specific reduction of *tRNA-Phe* abundance

**Fig. 2:**
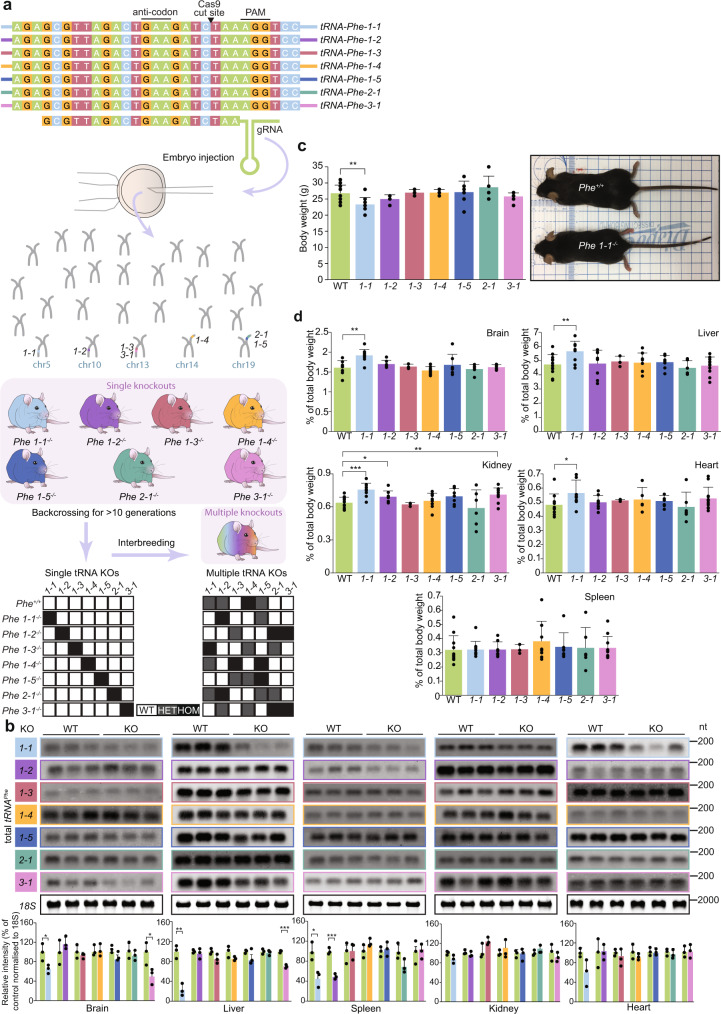
Tissue-specific distribution of *tRNA-Phe* isodecoders. **a** Schematic representing the seven mouse lines generated using a single CRISPR/Cas9 guide to knockout each of the *tRNA-Phe* genes in the mouse genome. The guide RNA was injected in mouse embryos to introduce deletions in each of the seven *tRNA-Phe* genes. Mice with *tRNA-Phe* gene deletions were identified by sequencing and individual *tRNA-Phe* gene deletions were backcrossed to wild-type mice to generate single *tRNA-Phe* gene deletions. The single *tRNA-Phe* gene deletion mice were bred for at least 10 generations before commencing molecular and phyiological experiments. Single *tRNA-Phe* gene deletions were interbred to generate multiple *tRNA-Phe* gene deletions. **b** The abundance of total *tRNA-Phe* was measured by northern blotting in brain, liver, spleen, kidney and heart, in control and *tRNA-Phe* knockout mice for each of the seven *tRNA-Phe* knockout lines in at least three independent biological experiments with similar results. 18S rRNA was used as a loading control, and one representative blot is shown for each tissue. All values presented in panel **b** are means ± SD of *n* = 3. *p < 0.05, **p < 0.01, ***p < 0.001, Student’s two-way *t*-test (*p* = 0.039 for brain *tRNA-Phe-1-1*, *p* = 0.026 for brain *tRNA-Phe-3-1*, *p* = 0.0017 for liver *tRNA-Phe-1-1*, *p* = 0.017 for spleen *tRNA-Phe-1-1* and *p* = 0.00048 for spleen *tRNA-Phe-1-2*). **c** Body weight differences between control and *tRNA-Phe* knockout male mice at 10 weeks of age. All values presented in panel **c** are means ± SD of *n* = 4. **p < 0.01, Student’s two-way *t*-test (*p* = 0.0011 for *tRNA-Phe-1-1*). Photo illustrates the typical size differences between wild-type (WT) and *tRNA-Phe-1-1* knockout mice. **d** Tissue weight-to-body weight ratio of brain, liver, spleen, kidney and heart in control and *tRNA-Phe* knockout male mice at 10 weeks of age, values are means ± SD (*n* = 4); Student’s two-way *t*-test (*p* = 0.001 for brain *tRNA-Phe-1-1*, *p* = 0.0055 for liver *tRNA-Phe-1-1*, *p* = 0.000061 for kidney *tRNA-Phe-1-1*, *p* = 0.032 for kidney *tRNA-Phe-1-2*, *p* = 0.005 for kidney *tRNA-Phe-3-1*, and *p* = 0.041 for heart *tRNA-Phe-1-1*).

We used CRISPR-Cas9 genome editing to delete the *tRNA-Phe* family of genes in mice and bred the mice with deleted genes to homozygosity for each *tRNA-Phe* gene. We generated seven homozygous single *tRNA-Phe* gene deletion knockout mouse strains: *tRNA-Phe-1-1*^*-/-*^, *tRNA-Phe-1-2*^*-/-*^*, tRNA-Phe-1-3*^*-/-*^*, tRNA-Phe-1-4*^*-/-*^*, tRNA-Phe-1-5*^*-/-*^*, tRNA-Phe-2-1*^*-/-*^ and *tRNA-Phe-3-1*^*-/-*^ (Fig. 2a) and validated their specific deletion by Sanger sequencing (Supplementary Fig. 2). We investigated how the single *tRNA-Phe* gene deletions affected *tRNA-Phe* levels in brain, liver, spleen, kidney and heart in each line by northern blotting. We show that the loss of *tRNA-Phe-1-1*^*-/-*^ caused the most pronounced reduction in total *tRNA-Phe* levels in the liver and brain compared to control mice (Fig. 2b), consistent with the specific deletion of the *tRNA-Phe-1-1*^*-/-*^ gene, validated by whole-genome sequencing (Supplementary Data 1). In addition, the loss of *tRNA-Phe-3-1*^*-/-*^ resulted in specific reduction of *tRNA-Phe* levels in the liver and brain and loss of *tRNA-Phe-1-2* caused a reduction of total *tRNA-Phe* levels in the spleen, whereas the loss of the remaining *tRNA-Phe* genes did not notably affect the total *tRNA-Phe* content in the five different tissues of each line compared to control mice (Fig. 2b). Next, we investigated the molecular and physiological consequences of each single gene loss compared to age- and sex-matched control littermates. The body weights of the single gene knockout mice were not different to the control mice with the exception of the significantly reduced weight of the *tRNA-Phe-1-1*^*-/-*^ mice (Fig. 2c). The brain, liver and kidney weight relative to the body size was increased in the *tRNAPhe-1-1*^*-/-*^ mice, and kidney weight was also increased in the *tRNA-Phe-1-2*^*-/-*^ and *tRNA-Phe-3-1*^*-/-*^ mice (Fig. 2d). Analyses of the blood profiles of the seven knockout lines compared to control mice revealed reduction in white blood cells, lymphocytes and haemoglobin and increase in red cell distribution width and mean platelet volume in the *tRNA-Phe-1-1*^*-/-*^ mice (Supplementary Fig. 3). Other knockout lines, including *tRNA-Phe-1-2*^*-/-*^ and *tRNA-Phe-1-3*^*-/-*^ mice, also had reduced white blood cells, neutrophils, lymphocytes, red blood cells, haemoglobin, haematocrit and increased platelet count, mean corpuscular haemoglobin and red cell distribution width (Supplementary Fig. 3), although these changes did not manifest in overt phenotypes in the knockout mice that differed from the control mice. These findings suggest that the multi-copy nature of *tRNA-Phe* genes may compensate for the loss of a single gene from the family with the exception of the *tRNAPhe-1-1*^*-/-*^ and *tRNAPhe-3-1*^*-/-*^ in specific tissues such as the brain and liver, indicating tissue-specific requirements for these genes to maintain the pool of *tRNA-Phe* in mammals.

### Transcriptome- and proteome-wide analyses reveal neurological defects in the *tRNA-Phe1-1*^*-/-*^ mice

**Fig. 3:**
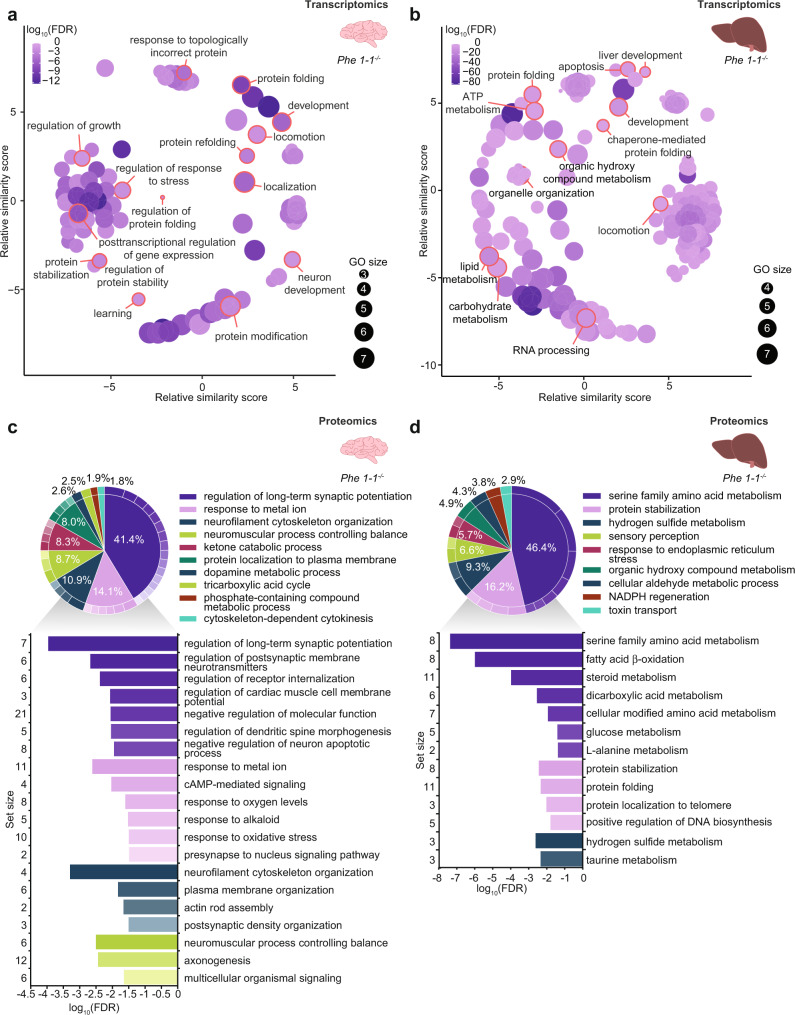
Transcriptome and proteome-wide molecular signatures of neurological defects in the absence of *tRNA-Phe-1-1* genes in the brain. Transcriptome-wide changes in brains **a**, and livers **b**, from *tRNA-Phe-1-1*^*-/-*^ knockout mice (*n* = 3) compared to controls (*n* = 3), summarised by biological process gene ontologies (GOs) determined by PANTHER and visualised using REVIGO. GO terms have been summarised and grouped by REVIGOs internal clustering algorithm. GO size represents the number of total genes in each specific ontology, the colour scale represents the degree of significance and parent GO terms are marked with a red outline. Brain **c**, and liver **d**, proteomes of *tRNA-Phe-1-1*^*-/-*^ knockout mice (*n* = 5) compared to controls (*n* = 5), summarised as biological process GOs determined by PANTHER and REVIGO and visualised with CirGO. GO terms have been summarised and grouped by REVIGO’s internal clustering algorithm. The pie-charts display the proportion of genes changing from each GO category as generated by CirGO with proportions indicated and top GO terms that are collapsed within each representative term are shown as a bar graph with log10(FDR) and gene set size for each ontology.

We selected the knockout lines *tRNA-Phe-1-1*^*-/-*^, *tRNA-Phe-2-1*^*-/-*^ and *tRNA-Phe-3-1*^*-/-*^ as representative genes for each isodecoder within the *tRNA-Phe-GAA* family and because we identified the greatest reduction in total *tRNA-Phe* levels in the *tRNA-Phe-1-1*^*-/-*^ and *tRNA-Phe-3-1*^*-/-*^ mice compared to the other *tRNA-Phe* knockout and control mice. We carried out RNA sequencing to globally profile the transcriptome-wide changes in the brains and livers of the *tRNA-Phe-1-1*^*-/-*^, *tRNA-Phe-2-1*^*-/-*^ and *tRNA-Phe-3-1*^*-/-*^ mice compared to control mice (Fig. 3a, b, Supplementary Fig. 4a–c and Supplementary Fig. 5a, b). We identified 475 downregulated and 691 upregulated genes in the brain of the *tRNA-Phe-1-1*^*-/-*^ mice that revealed predominantly reduced neuronal transcriptional activity and increased requirement for protein folding and chaperone function (Fig. 3a, Supplementary Data 2 and Supplementary Fig. 5a). In contrast, there were very minimal transcriptional changes in the brains from the *tRNA-Phe-2-1*^*-/-*^ mice (Supplementary Data 2 and Supplementary Fig. 5a) that affected genes involved in leucocyte and hemopoiesis regulation, reflecting the observed reduction in the lymphocyte, monocyte and red blood cell numbers in the *tRNA-Phe-2-1*^*-/-*^ mice (Supplementary Fig. 3). In the brains of the *tRNA-Phe-3-1*^*-/-*^ mice we identified a greater number of changes compared to the control and *tRNA-Phe-2-1*^*-/-*^ mice, indicative of neuronal signalling and immune responses in the absence of *tRNA-Phe-3-1* (Supplementary Fig. 4a and Supplementary Fig. 5a). The transcriptional changes in the livers of the *tRNA-Phe-1-1*^*-/-*^ mice revealed reduction in lipid metabolic pathways and increased protein folding responses (Fig. 3b, Supplementary Data 2 and Supplementary Fig. 5b), that were also observed in the brain. Reduction in lipid metabolic pathways was also found in the livers of the *tRNA-Phe-2-1*^*-/-*^ and *tRNA-Phe-3-1*^*-/-*^ mice (Supplementary Fig. 4b, c and Supplementary Fig. 5b), and an increase in mitosis and cell proliferation was predominant in the livers of the *tRNA-Phe-3-1* (Supplementary Fig. 4c and Supplementary Fig. 5b), possibly acting to promote the regenerative capacity of the liver. These analyses reveal that despite similar reduction of *tRNA-Phe* levels upon single loss of either *tRNAPhe-1-1* or *tRNAPhe-3-1*, the transcriptional consequences of their loss are different, suggesting that different tRNA genes are not entirely redundant, either as a consequence of tissue-specific requirements for specific tRNA genes or the possible divergence of isodecoder function.

Proteome-wide changes in the brains and livers of *tRNA-Phe-1-1*^*-/-*^, *tRNA-Phe-2-1*^*-/-*^, *tRNA-Phe-3-1*^*-/-*^ and control mice were investigated by quantitative mass spectrometry (Fig. 3c, d and Supplementary Fig. 6a, b) identified 170 and 128 significantly changing proteins in brain and liver, respectively. The most prominent changes were found in the proteomes from the *tRNA-Phe-1-1*^*-/-*^ brains that revealed the greatest changes in synaptic potentiation followed by ion signalling, indicating neurological impairment at the protein level consistent with the observed transcriptional changes (Fig. 3c). Calretinin (CALB2) was significantly reduced in brain proteomes of the *tRNA-Phe-1-1*^*-/-*^ mice, as well as neurocalcin delta (NCALD) (Supplementary Fig. 7a and Supplementary Data 3), both of which are involved in neuronal and calcium signalling in the brain. The proteome changes in the brains of the *tRNA-Phe-2-1*^*-/-*^ and *tRNA-Phe-3-1*^*-/-*^ mice highlighted processes involved in amino acid metabolism, protein and ion transport related to neuronal signalling (Supplementary Fig. 6a, Supplementary Fig. 7a and Supplementary Data 3). The liver proteomes revealed the greatest changes in protein folding (protein disulfade isomerases, HSP90), amino acid metabolism (amino acid transferases) and fatty acid oxidation (fatty acid synthase, acetyl-coenzyme A thioesterase 1, acetyl-CoA dehydrogenases) in the *tRNA-Phe-1-1*^*-/-*^ mice (Fig. 3d, Supplementary Figs. 6b and 7b and Supplementary Data 3), compared to changes in lipid metabolism in the *tRNA-Phe-2-1*^*-/-*^ mice and protein synthesis and carbohydrate metabolism in *tRNA-Phe-3-1*^*-/-*^ mice, respectively (Supplementary Figs. 6b and 7b). Taken together these analyses reveal that the loss of *tRNA-Phe-1-1* results in the greatest transcriptional and proteomic perturbations, indicative of a neurological defect in these mice.

### The loss of *tRNA-Phe-1-1* causes reduction in calbindin levels in Purkinje cells leading to neurological and behavioural changes

**Fig. 4:**
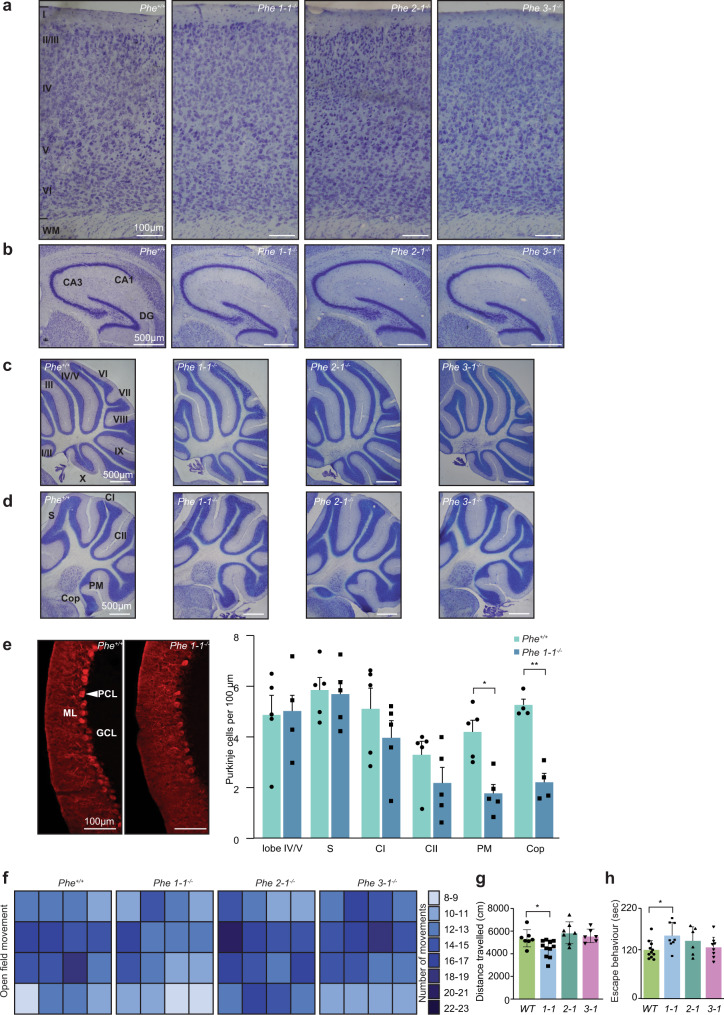
Effect of *tRNA-Phe* gene loss on brain morphology and behaviour in 10-week old mice. **a**, **b** Sagittal sections of the brain in control (*Phe*^*+/+*^), *tRNA-Phe-1-1*^*-/-*^, *tRNA-Phe-2-1*^*-/-*^ and *tRNA-Phe-3-1*^*-/-*^ mice (*n* = 7 of each genotype) were stained with toluidine blue and assessed for histological changes in the cortex **a**, Cortex (pia is at the top), labels in the first panel show layers I to VI and the white matter (WM). **b** Hippocampus (DG: dentate gyrus, CA: cornu ammonis (hippocampus) regions). **c** vermis of the cerebellum. **d** hemisphere of the cerebellum. **e** Immunostaining with calbindin in sections from the hemisphere of the cerebellum (paramedian: PM lobe shown) revealed a decrease in calbindin-positive Purkinje cells in the *tRNA-Phe1-1* knockout mice compared to controls. All values are means ± SEM of *n* = 5 *p < 0.05, **p < 0.01, *(p* = 0.0034 for PM and *p* = 0.00066 for the copula of the pyramis: Cop), two-way, unpaired, Student’s *t*-test; ML molecular layer, PCL Purkinje cell layer (indicated with a white arrowhead), GCL granule cell layer, C cortex layers, S subiculum. All experiments shown in **a**–**e** were repeated independently up to seven times with similar results. **f** Heatmaps representing the layout of the open field testing area show the overall movement of control (*Phe*^*+/+*^), *tRNA-Phe-1-1*^*-/-*^, *tRNA-Phe-2-1*^*-/-*^ and *tRNA-Phe-3-1*^*-/-*^ mice (*n* = 7 of each genotype). Shading represents the number of times a box was entered over a 10-min testing period. **g** The distance travelled by the control (*Phe*^*+/+*^), *tRNA-Phe-1-1*^*-/-*^, *tRNA-Phe-2-1*^*-/-*^ and *tRNA-Phe-3-1*^*-/-*^ mice was measured using DeepLabCut (*n* = 7), values are means ± SD of *n* = 7 *p < 0.05, two-way Student’s *t*-test and one-way ANOVA (*p* = 0.025 for *tRNA-Phe-1-1*). **h** Duration of escape-oriented behaviour recorded over a 5 min testing period in control and knockout mice during the tail suspension test. All values are means ± SD of *n* = 7 *p < 0.05, two-way Student’s *t*-test and one-way ANOVA (*p* = 0.012 for *tRNA-Phe-1-1*).

To investigate if reduction in *tRNA-Phe-1-1* levels may affect brain structure and morphology, as indicated by the transcriptomic and proteomic analyses, we performed toluidine-blue staining of sagittal brain sections in mice lacking this tRNA and compared them to the *tRNA-Phe-2-1*^*-/-*^, *tRNA-Phe-3-1*^*-/-*^ and wild-type mice. We observed no structural changes in the cortex, hippocampus or cerebellum of knockout mice compared to controls (Fig. 4a–d), indicating that by 10 weeks the *tRNA-Phe-1-1* knockout had not caused gross changes in brain structure. Neurological changes occurred only at a cellular level, as suggested by the proteomic and transcriptomic data. Immunostaining revealed a decrease in calbindin-positive Purkinje cells throughout the cerebellum, which was most significant in specific lobules of the cerebellar hemispheres in *tRNA-Phe-1-1*^*-/-*^ compared to the control mice (Fig. 4e and Supplementary Fig. 8a).

Behavioural assessments using an open field test revealed reduced movement and changes in the exploratory behaviour of the *tRNA-Phe-1-1*^*-/-*^ mice compared to controls, and to a lesser degree in the *tRNA-Phe-3-1*^*-/-*^ mice (Fig. 4f and Supplementary Fig. 8b). We quantitated the movement of the mice and found that the distance travelled by the *tRNA-Phe-1-1*^*-/-*^ mice was significantly reduced compared to controls (Fig. 4g). The tail suspension test showed that the *tRNA-Phe-1-1*^*-/-*^ mice had increased escape behaviour compared to the control mice (Fig. 4h), which was also reflected by the balance beam test where the *tRNA-Phe-1-1*^*-/-*^ mice were faster than the control, *tRNA-Phe-2-1*^*-/-*^ and *tRNA-Phe-3-1*^*-/-*^ mice, who had a significant delay in crossing speed, indicative of potential motor coordination defects (Supplementary Fig. 8b). The *tRNA-Phe-1-1*^*-/-*^ mice did not have any visual impairment compared to the control mice (Supplementary Fig. 8b), suggesting that the behavioural changes of the knockout mice were consistent with functional deficits in the cerebellum due to reduced calbindin levels in the Purkinje cells and related neurotransmitters identified in the transcriptomic and proteomic analyses (Figs. 3a, c and 4e). These findings reveal that a single isodecoder gene deletion can change behavioural patterns, and that specific tRNA isodecoder gene functions are not fully redundant.

Hematoxylin and eosin staining of the livers from the *tRNA-Phe-1-1*^*-/-*^ mice revealed centrilobular congestion and necrosis, while the livers from the *tRNA-Phe-2-1*^*-/-*^ and *tRNA-Phe-3-1*^*-/-*^ mice were comparable to those of control mice (Supplementary Fig. 8c). Centrilobular congestion is found in patients with hepatic venous outflow obstruction that prevents the drainage of blood from the liver resulting in liver enlargement^21^, which was consistent with the increased liver weight of the *tRNA-Phe-1-1*^*-/-*^ mice (Fig. 2c) and perturbed metabolic molecular profiles of the *tRNA-Phe-1-1*^*-/-*^ livers (Fig. 4b, d).

### The loss of *tRNA-Phe1-1* stimulates compensatory expression of other tRNAs

**Fig. 5:**
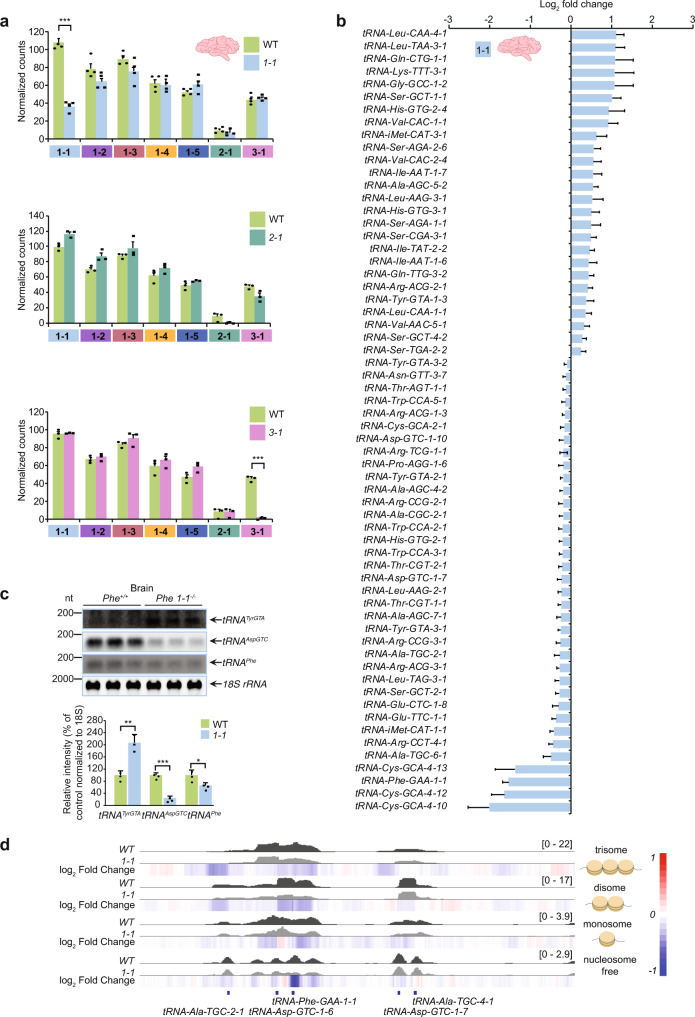
In the absence of *tRNA-Phe-1-1*, translation is compensated by increased expression of other tRNAs. **a** ATAC-Seq was used to identify changes in chromatin accessibility (normalised counts) of the seven *tRNA-Phe* genes in control mice compared to *tRNA-Phe-1-1*^*-/-*^, *tRNA-Phe-2-1*^*-/-*^ and *tRNA-Phe-3-1*^*-/-*^ knockout mice in brain. Results show the mean score for each set of replicates (*n* = 3 of each genotype) over *tRNA-Phe* gene regions (in different colour) for each of the three knockout mouse lines compared to control mice in brain; all values are means ± SEM. ***p < 0.001 (*p* = 0.000045 for *tRNA-Phe-1-1* locus in the *tRNA-Phe-1-1*^*-/-*^ mice, and *p* = 0.0006 for *tRNA-Phe-3-1* locus in the *tRNA-Phe-3-1*^*-/-*^ mice), Student’s two-way *t*-test. **b** Significant changes in chromatin accessibility of tRNA genes in brains of *tRNA-Phe-1-1*^*-/-*^ mice compared to controls, determined using ATAC-Seq; values are log_2_ fold changes ±SEM (*n* = 3 of each genotype), and all the significant changes determined using DESeq2 are shown. **c** Northern blotting of altered tRNAs in brains of *tRNA-Phe-1-1*^*-/-*^ mice compared to controls (*n* = 3 of each genotype); values are means ± SD; Student’s two-way *t*-test (*p* = 0.001 for *tRNA-Tyr*, *p* = 0.0055 for *tRNA-Asp*, and *p* = 0.041 for *tRNA-Phe*). **d** ATAC-Seq tracks show changes in the accessibility of nucleosome-free, mono-, di- and trinucleosome-bound chromatin in the brain within the *tRNA-Phe-1-1* locus of *tRNA-Phe-1-1*^*-/-*^ mice compared to controls. Coverage tracks are expressed in reads per million (RPM) and heatmap tracks show log_2_ fold change.

We used ATAC-Seq to determine the effects of single *tRNA-Phe-1-1*, *tRNA-Phe-2-1* and *tRNA-Phe-3-1* deletion on the chromatin accessibility of the remaining six *tRNA-Phe* alleles. We show that loss of each of the three *tRNA-Phe* alleles: *tRNA-Phe-1-1*, *tRNA-Phe-2-1* and *tRNA-Phe-3-1* in the brain only reduced the nucleosome-free chromatin of the specifically knocked out *tRNA-Phe* gene but did not affect the chromatin structure of the remaining six *tRNA-Phe* genes (Fig. 5a and Supplementary Figs. 9a and  10a–c). The sum of reads across all *tRNA-Phe* loci revealed that the loss of *tRNA-Phe-1-1* cannot be compensated by the presence of the other isodecoders, (Supplementary Fig. 10a), providing further support for the importance of this tRNA in the brain. In the liver, we also found that the deletion of *tRNA-Phe-1-1*, *tRNA-Phe-2-1* and *tRNA-Phe-3-1* specifically reduced their chromatin accessibility in each of the knockout mouse lines (Supplementary Figs. 9b and  11a).

To investigate compensatory changes in global tRNA expression in response to loss of *tRNA-Phe*, we examined the chromatin accessibility of all other tRNAs across the genome in each of the *tRNA-Phe-1-1*, *tRNA-Phe-2-1* and *tRNA-Phe-3-1* deletion strains. In the brain of the *tRNA-Phe-1-1*^*-/-*^ mice we identified marked reduction in nucleosome-free chromatin at the *tRNA-Phe-1-1* locus, in addition to significant increases in the expression of many other tRNA genes, which were upregulated in response to the loss of this tRNA gene (Fig. 5b). Specifically, we identified the highest increases in chromatin accessibility of *tRNA-Leu-CAA-4-1* and *tRNA-Leu-TAA-3-1*. Interestingly, we identified reduced chromatin accessibility in three genes from the *tRNA-Cys-GCA-4* isodecoder family along with the *tRNA-Phe-1-1* locus (Fig. 5b). We confirmed that the expression of specific tRNA decoders was consistent with the ATAC-Seq findings in the brains of the *tRNA-Phe-1-1*^*-/-*^ mice compared to control mice by northern blotting (Fig. 5c). Reduction of the nucleosome-free chromatin was the most pronounced in the brains of the *tRNA-Phe-1-1*^*-/-*^ mice compared to controls (Fig. 5d), but was not observed for mono-, di- or trinucleosome-bound chromatin. There were no increases in accessiblity at other tRNAs in the brains of the *tRNA-Phe-2-1*^*-/-*^ and *tRNA-Phe-3-1*^*-/-*^ mice compared to controls (Supplementary Fig. 10b, c), consistent with the lack of apparent phenotypic changes in these mice. In the livers of the *tRNAPhe-1-1*^*-/-*^ mice we also observed significant changes in other tRNA genes (Supplementary Fig. 11b) that were confirmed by northern blotting (Supplementary Fig. 11c). In the livers, the *tRNA-Phe-1-1*^*-/-*^ deletion markedly affected the surrounding chromatin beyond the *tRNA-Phe* locus, causing greater reduction in nucleosome-free coverage than at the site of the deletion itself (Supplementary Fig. 11d). In addition, mono- and tri-nucleosome-bound regions associated with these compacted regions were also reduced, as well as the di-nucleosome region between the tRNAs (Supplementary Fig. 11d). A mono-nucleosome bound region upstream and closely associated with *tRNA-Ala-TGC-2-1* was also more compact in the livers of the *tRNA-Phe-1-1*^*-/-*^ mice, and a noticeable reduction in nucleosome-free coverage downstream was associated with the *tRNA-Asp/Ala* loci. These data indicate that the expression of *tRNA-Phe-1-1* is important to facilitate the openness of surrounding chromosomal regions and to prevent loss of their expression via heterochromatinization. Furthermore, there are tissue-specific programmes that attempt to compensate for loss of *tRNA-Phe* expression, either by upregulation of other *tRNA-Phe* genes, as observed in the liver, or by increased transcription of specific tRNAs that carry other amino acids, as observed in both the brain and liver tissues.

**Fig. 6:**
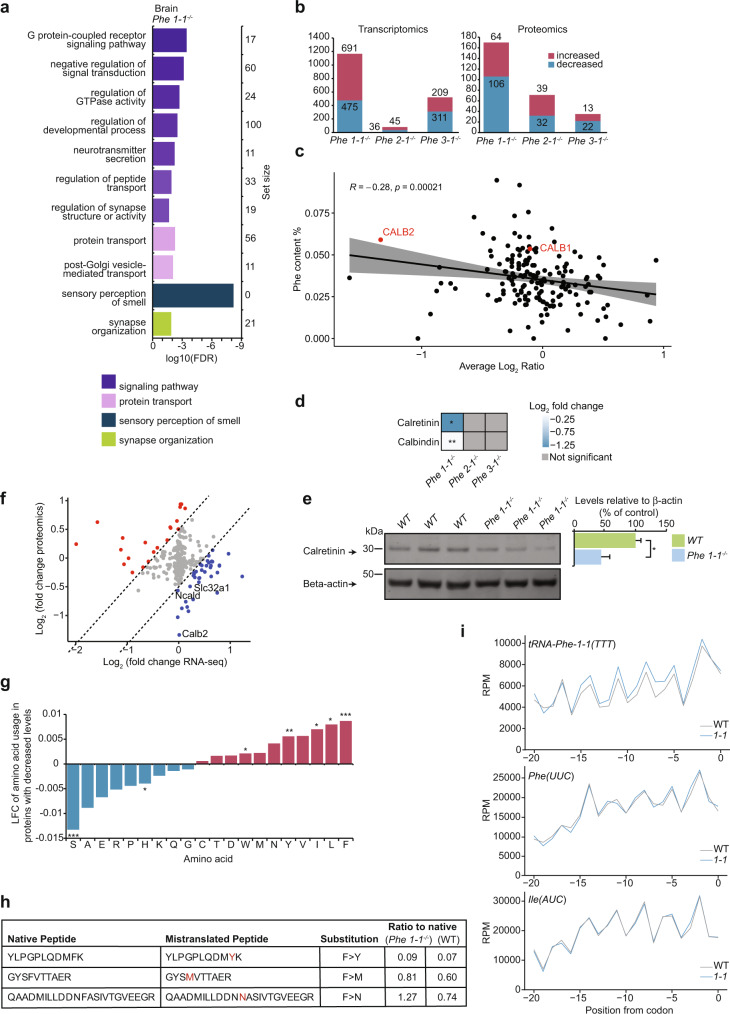
Amino acid misincorporation reduces the abundance of proteins with high phenylalanine content. **a** Biological processes most affected in ATAC-Seq of *tRNA-Phe-1-1*^*-/-*^ mouse brains compared to controls, summarised using PANTHER and REVIGO. **b** Transcriptomic and proteomic changes in *tRNA-Phe-1-1*^*-/-*^ mouse brains. Red indicates significant (*s* < 0.01 determined by DESeq2, adj *p* < 0.05 determined using Spectronaut) log_2_-fold increases compared to the control and blue indicates decreases. Numbers of transcripts or peptides changed are indicated. **c** Comparison of phenylalanine usage in proteins compared to their change in abundance in the brain proteomes of *tRNA-Phe-1-1*^*-/-*^ mice relative to controls. Percentage of phenylalanine content in each detected protein is plotted against average log_2_ ratios± SD (grey) in brain proteomes from *tRNA-Phe-1-1*^*-/-*^ mice; calbindin and calretinin are highlighted in red. Spearman’s rank correlation coefficient and *p*-value are shown. **d** Calbindin and calretinin levels in brains of *tRNA-Phe****-****1-1*^*-/-*^ mice (*n* = 5), determined by Spectronaut (*adj *p* = 0.026, **adj *p* = 0.0053). **e** Immunoblotting of calretinin in *tRNA-Phe-1-1*^*-/-*^ and control brain homogenates (*n* = 3), quantitated relative to β-actin. *p* = 0.027, Student’s two-way *t*-test, values are means ± SD. **f** Comparison of the common, significantly changing proteins (adj *p*-value < 0.05) and transcripts (adj *p*-value < 0.05) in the brain *tRNA-Phe-1-1*^*-/-*^ mice relative to controls. **g** Amino acid content in proteins that are reduced in brains of *tRNA-Phe-1-1*^*-/-*^ mice identified by mass spectrometry. Amino acids that occur at a higher frequency in proteins reduced in *tRNA-Phe-1-1*^*-/-*^ mice are in red, amino acids with lower lower occurrence are in blue; S *p* = 0.00041, H *p* = 0.016, W *p* = 0.044, Y *p* = 0.0025, I *p* = 0.021, L *p* = 0.049, F *p* = 0.00022. Student’s two-way *t*-test was used for **c**, **f**, **g**. **h** Mistranslated peptides identified in the brain proteomes of *tRNA-Phe-1-1*^*-/-*^ mice and their ratio relative to their canonical peptides in WT and *tRNA-Phe-1-1*^*-/-*^ mice. **i** Metagene analysis of averaged ribo-profil**i**ng in control and *tRNA-Phe-1-1*^*-/-*^ mice (*n* = 3), aligned to Phe (UUU), *Phe* (UUC) codons and an unrelated Ile (AUC) codon shows a specific increase in aligned read counts upstream of Phe (UUU) codons, and indicates ribosomal stalling at the A, P and E sites in the brains of *tRNA-Phe-1-1*^*-/-*^ mice.

Changes in chromatin accessibility of mRNA-encoding genes were detected in *tRNA-Phe* knockout mice (Fig. 6a and Supplementary Fig. 10). The greatest changes were in the brains of the *tRNA-Phe-1-1*^*-/-*^ mice, affecting genes involved in signalling, protein transport, sensory perception of smell and synapse organisation (Fig. 6a). In *tRNA-Phe-2-1*^*-/-*^ brains, similar gene ontologies were detected (Supplementary Fig. 12a), while *tRNA-Phe-3-1*^*-/-*^ knockout mice had too few changes in mRNA-encoding genes for summarisation. These results reflect the observed phenotypes of these mice, with *tRNA-Phe-1-1*^*-/-*^ having significant decreases in brain and body size, compared to *tRNA-Phe-2-1*^-/-^ and *tRNA-Phe-3-1*^*-/-*^ that had no apparent phenotype. Changes in the livers of the *tRNA-Phe* mice were observed in genes involved in signalling pathways, protein modifications and metabolism (Supplementary Fig. 12b–d). The differences between brain and liver indicate that the requirements for tRNAs in these tissues differ and highlight the broad range of responses that can occur between tissues.

### Loss of *tRNA-Phe-1-1* reduces the levels of proteins high in phenylalanine in the brain

Altered tRNA abundance has been suggested to selectively modulate the translation of specific transcripts to vary proteome composition during stress^22^. Therefore, we investigated the amino acid content of significantly changing proteins identified by mass spectrometry analyses (Fig. 6b) in the brain proteomes from the control and *tRNA-Phe-1-1*^*-/-*^ mice and found that there was a correlation between phenylalanine content and protein expression in the knockouts (Fig. 6c). Proteins high in phenylalanine content were reduced, while proteins low in phenylalanine content increased (Fig. 6c). It is likely that proteins high in phenylalanine are unable to be upregulated as effectively due to the restricted *tRNA-Phe* abundance, limiting the incorporation of phenylalanine into proteins during translation. Notable affected proteins include the two homologous calcium-binding proteins that are important for neuronal function, calbindin (CALB1) and calretinin (CALB2). Both proteins have a high phenylalanine content compared to the average of the mouse proteome and are specifically down-regulated in *tRNA-Phe-1-1*^*-/-*^ knockout brains (Fig. 6d and Supplementary Fig. 13). In addition, immunoblotting confirmed the significant reduction of calretinin in these mice (Fig. 6e). Additional proteins with high phenylalanine content, including NCALD, SLC32A1 and CAPZA1, were also reduced, that are involved in calcium binding (NCALD and CAPZA1) or as solute carriers involved in transport to synaptic vesicles (SLC32A1) (Supplementary Fig. 13). Calcium has major roles in neurons as it triggers neurotransmitter release, and therefore the decreased abundance of many calcium-binding proteins may affect neurotransmission in the *tRNA-Phe-1-1*^*-/-*^ knockout mice, consistent with our RNA-seq findings (Fig. 3a), reduced calbindin levels in Purkinje cells (Fig. 4e) and behavioural changes in these mice (Fig. 4f, g).

The decreased availability of *tRNA-Phe* in *tRNA-Phe*-*1-1*^*-/-*^ mice appears to directly impact the ability of the cells to properly translate a proportion of mRNAs to proteins, and the abundance of many proteins was significantly altered without concomitant changes in their encoding mRNAs (Fig. 6f). As the reduced availability of *tRNA-Phe* appears to hinder the effective incorporation of phenylalanine into the growing peptide chain during translation, we investigated amino acid usage in the up- and down-regulated *tRNA-Phe*-*1-1*^*-/-*^ proteomic results (Fig. 6g). We found that significantly more phenylalanine codons are observed in the down-regulated proteins, in addition to leucine, isoleucine, tyrosine and tryptophan. These amino acids have codons that are similar to phenylalanine and may be reduced in upregulated proteins as mistranslation involving their corresponding tRNAs due to the reduction of *tRNA-Phe* may result in less tRNAs available, consistent with the changes in other tRNAs (Fig. 5b). Analyses of the brain proteomes in *tRNA-Phe-1-1*^*-/-*^ and control mice mice identified examples of peptides with apparent increased misincorporation of specific amino acids in place of phenylalanine (Fig. 6h and Supplementary Fig. 14), confirming that the fidelity of translation can be affected with the specific deletion of *tRNA-Phe* genes in these mice. This finding is consistent with the increased levels of other tRNAs, some with near-cognate anticodons to *tRNA-Phe*, suggesting that their upregulation can compete for phenylalanine codons in the absence of *tRNA-Phe-1-1*. We carried out ribosome profiling (ribo-profiling) to investigate how the loss of the *tRNA-Phe-1-1* gene affects translation in the brain (Fig. 6i and Supplementary Fig. 15) and found specific ribosome stalling at Phe (UUU) codons in the *tRNA-Phe-1-1*^*-/-*^ mice compared to controls (Fig. 6i). The ribosome profile of Phe (UUC) did not differ between the *tRNA-Phe-1-1*^*-/-*^ and control mice (Fig. 6j), likely due to the more efficient codon-anticodon interaction afforded by the G-C base pairing at the third position of the codon in contrast to the G-U pairing of the stalled Phe (UUU) codon^23^. Ribosome stalling was found in mRNAs encoding calbindin and calretinin as well as calcium binding NCALD and solute carrier SLC32A1 (Supplementary Fig. 15a, b) consistent with their reduced levels. Ribosome stalling was exacerbated in mRNAs with consecutive polyphenylalanine codons (Supplementary Fig. 15b), also involved in neurotransmission. Taken together these data show that loss of *tRNA-Phe-1-1* impacts the translation of mRNAs enriched in Phe codons.

### The number and identity of *tRNA-Phe* isodecoder alleles determine embryo and developmental viability

**Fig. 7:**
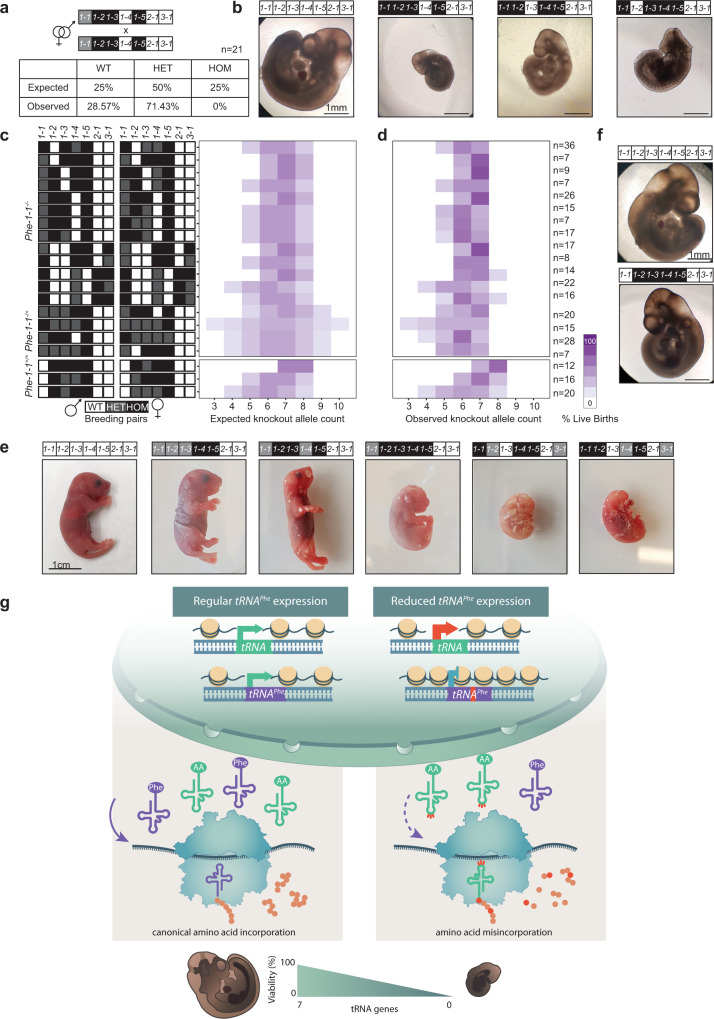
Hierarchical requirements of *tRNA-Phe* genes for embryonic viability and development. **a** Expected and observed Mendelian inheritance of gene loci homozygous for the deletion of up to four *tRNA-Phe* alleles including *tRNA-Phe-1-1*. Black denotes homozygous loss of an allele, grey heterozygous loss of one allele and white is indicative of two wild-type alleles. **b** Embryos with *tRNA-Phe-1-1*^*-/-*^ and three additional homozygous *tRNA-Phe* deletions were underdeveloped at day E10 when compared to controls. **c** Genotypes of breeding pairs set up to produce mice with multiple *tRNA-Phe* deletions, including either a heterozygous or homozygous *tRNA-Phe-1-1* deletion (black represents homozygous loss of the allele, grey is heterozygous loss of one allele and white is indicative of two wild-type alleles), and percentage of expected live births. **d** Observed percentage of live births (where white is no animals observed with this phenotype). The number of embryos examined are shown on the right of the heatmap. **e** Stillborn mice born at varied stages of development, with an increasing number of *tRNA-Phe* knockout alleles correlating to an earlier developmental failure. **f**, Normal development of E10 embryos with *tRNA-Phe-1-1*^*+/+*^ and *tRNA-Phe-1-2*^*-/-*^, *tRNA-Phe-1-3*^*-/-*^, *tRNA-Phe-1-4*^*-/-*^ and *tRNA-Phe-1-5*^*-/-*^ compared to controls. **g** Canonical amino acid incorporation takes place during translation in wild-type mice expressing *tRNA-Phe* genes that maintain chromatin openness. Deletion of the dominant *tRNA-Phe-1-1* allele, causes chromatin condensation at the *tRNA-Phe* allele and leads to amino acid misincorporation that reduces the stability of specific proteins required for neurological function and development. Sequential deletion of seven or more *tRNA-Phe* alleles reduces embryo viability and survival of mice.

We generated mice with multiple *tRNA-Phe* deletions to identify which genes were most essential for survival, and how many *tRNA-Phe* alleles were required for survival. We intercrossed *tRNA-Phe* deletion strains repeatedly, at each stage selecting mice with the greatest numbers of deletion alleles for subsequent breeding. When genotyping the offspring from one of these matings we observed that when *tRNA-Phe-1-2*, *tRNA-Phe-1-3* and *tRNA-Phe-1-5* were homozygously deleted, we never observed homozygous knockouts of *tRNA-Phe-1-1*, despite Mendelian predictions (Fig. 7a). To confirm these observations, we performed timed matings and dissected embryos at day 10 (E10). We observed that embryos with four homozygous *tRNA-Phe* knockouts (including the *tRNA-Phe-1-1* gene) were underdeveloped compared to wild-type embryos at the same time point (Fig. 7b). Therefore, between E10 and birth these embryos fail to develop correctly and are either resorbed or stillborn.

Extensive subsequent matings with mice deleted in multiple *tRNA-Phe* alleles identified that it is possible to knock out a maximum of seven *tRNA-Phe* alleles, when the *tRNAPhe-1-1* gene is either fully (*tRNA-Phe-1-1*^*-/-*^) or partially (*tRNA-Phe-1-1*^*+/-*^) knocked out (Fig. 7c). We determined this by setting up breeding pairs with the capacity to produce offspring with up to eight or nine knockout alleles, but only observed live mice with a maximum of seven *tRNA-Phe* knockout alleles. The exception to this were mice with a homozygous *tRNA-Phe-1-1* deletion where we were able to knockout eight alleles only if one of the knocked alleles was *tRNA-Phe-2-1* (Fig. 7d), that we have shown has negligible expression (Fig. 1f and Supplementary Fig. 1c, d), indicating that this gene may not be functionally necessary for development. Interestingly, over the course of these breeding experiments we recorded numerous stillbirths and observed a trend where pups were increasingly underdeveloped with an increasing number of deleted *tRNA-Phe* alleles (Fig. 7e). However, adding a further level of complexity to this trend, we also noted that the level of underdevelopment was more severe in stillborn mice with eight knockout alleles that included a homozygous *tRNA-Phe-1-1* deletion (Fig. 7e, fifth and sixth image), compared to mice with nine knockout alleles that were only heterozygous for the *tRNA-Phe-1-1* deletion (third image). This indicates that the *tRNA-Phe-1-1* gene is the most critical for development. We bred mice with a wild-type *tRNA-Phe-1-1* gene and four additional homozygous knockouts (or eight alleles) to determine if it was possible to knockout more than seven *tRNA-Phe* alleles if the *tRNA-Phe-1-1* gene was intact. We found viable embryos whose development was comparable to wild-type embryos at E10 (Fig. 7f), and also found that a wild-type *tRNA-Phe-1-1* gene and four additional homozygous knockouts (or eight alleles) can result in viable mice that survived without health concerns beyond 10-weeks of age. This reveals that when the *tRNA-Phe-1-1* gene is either partially or fully deleted, a combination of up to seven alleles is compatible with life, however, a greater number of deletions are possible if the *tRNA-Phe-1-1* gene is not compromised, indicating that the *tRNA-Phe-1-1* gene is the most critical *tRNA-Phe* family member for development and survival.

## Discussion

Genome-wide analyses of the consequences of mammalian multigene tRNA losses or mutations in vivo have not been explored previously. We addressed this knowledge gap by creating single and multiple deletions of the *tRNA-Phe* genes in mice to reveal the requirement for isodecoder tRNA genes in translation and their compatibility with development and survival. We reveal that although the abundance of isodecoder tRNAs is required to maintain protein homeostasis, specific single genes are not functionally redundant. The roles of specific isodecoder *tRNA-Phe* genes and the compensatory responses to their loss vary between tissues, highlighting the rationale for isodecoder gene expansion in mammals. Importantly, we show that within the *tRNA-Phe* family there is a requirement for the expression of at least eight alleles including the specific isodecoder gene, *tRNA-Phe-1-1*, for survival and early embryo development. These findings highlight the need to maintain high *tRNA-Phe* levels and the hierarchical importance of different isodecoder tRNA genes for organismal function.

Mutations in tRNAs encoded by the mitochondrial genome are responsible for the majority of mitochondrial diseases and have been explored in detail^24^, however, unlike nuclear tRNAs each of these is present as only a single gene. The multi-copy nature of nuclear tRNAs likely buffers them from such dramatic losses of function but advances in whole-genome sequencing may provide new avenues to explore defects in nuclear tRNAs in human disease. Recently, compound heterozygous mutations in the gene encoding the Phe-tRNA synthetase, that aminoacylates *tRNA-Phe*, were linked to multi-system pulmonary disease, albeit without any evident impact on protein synthesis^25^. A single nucleotide polymorphism in the stem of the T-loop of the nuclear encoded isodecoder *tRNA-Arg-UCU-4-1* (also known as *n-Tr20*) combined with the loss of a translational GTPase caused neurodegeneration due to ribosomal stalling^17^. The expression of the *tRNA-Arg-UCU-5* was specific to the central nervous system and the SNP caused a reduction in the total levels of this tRNA^17^ and reduced the seizure thresholds caused by defects in GABA signalling^26^, exemplifying the requirement of specific isodecoders within a tRNA family that cannot be complemented by other isodecoders. The importance of specific decoders within tRNA families may become more evident when their function is compromised in the brain, where high levels of translation and therefore tRNA are required, as we found with *tRNA-Phe-1-1,* where its deletion caused ribosome stalling resulting in neuronal and behavioural defects. Increased expression of tRNAs from two isoacceptor families for glutamine (*tRNA-Glu-UUC*) and arginine (*tRNA-Arg-CCG*), respectively, compete for translating ribosomes and the expression of metastatic mRNAs containing complementary codons^27^, indicating that single isoacceptors can have specific roles that can override the total load of tRNAs from the same family and alter cell metabolism.

It has been suggested that clusters of tRNA genes can act as barriers to separate chromatin domains^28^. The expression of tRNA gene clusters has been shown to be genome-wide and stable across different tissues in mammals^6,29,30^, and this was consistent with the *tRNA-Phe* genes that were constitutively expressed between different tissues, albeit *tRNA-Phe-2-1* had the lowest expression in brain and liver. Spatial clustering of specific tRNA families in the genome or the copy number, including isoacceptor frequency, and the local proximity to other tRNA- or protein-coding genes have been shown not to affect the expression of tRNAs, however, chromatin accessibility affects tRNA expression^29^, as we have found for *tRNA-Phe* in our datasets. Reduced chromatin accessibility in the three *tRNA-Phe* isodecoder genes that were deleted in the mice impacted the openness of surrounding regions and ultimately their expression. Therefore, tRNA genes that are located close to protein-coding genes may actively facilitate the accessibility of transcription factors and RNA polymerases to promote transcription.

Loss of single *tRNA-Phe* genes did not result in gross phenotypic effects in mice, similarly to the lack of fitness defects in yeast upon individual tRNA deletions^31^, where isodecoders or tRNAs with similar anticodons were able to compensate for the loss of single tRNA gene deletions. Although it has previously been suggested that complementation by isoacceptors or isodecoders could stabilise the levels of the same tRNA family in yeast^29,31^, we show that in mammals the expression of isodecoders or different tRNAs from other families can largely compensate for the deficit of a single tRNA gene deletion in mammals.

Despite the overall compensation observed upon loss of the different *tRNA-Phe* isodecoder genes, loss of the *tRNA-Phe-1-1* gene resulted in subtle defects in growth, organ size, neurological development and behaviour. At a molecular level, we observed alterations in gene expression and protein abundance. Interestingly, proteins with high phenylalanine content, such as calretinin, tended to be reduced in abundance and peptides with mistranslated phenylalanine codons were observed by mass spectrometry, in line with a deficit in *tRNA-Phe* availability for translation. As well as causing inefficient translation and miscoding^32^, too little of a tRNA for a particular amino acid could increase the cell’s GTP usage, as GTP is required for proofreading of near-cognate tRNA-codon interactions, which could reduce the pool of productive ribosomes and ultimately affect growth rate^33^. This is in line with the observed growth reduction in the *tRNA-Phe-1-1* knockouts. Furthermore, as not all mRNAs are affected equally when translation is depressed^34^, specific molecular and phenotypic defects might be observed, such as the neurological changes in the *tRNA-Phe-1-1* knockout mice.

An outstanding question in the tRNA biology field is if the different isodecoders found in isoacceptor families are functionally identical, or simply a result of gene duplication and neutral mutation. Previous work has shown that differences within the tRNA body can alter their aminoacylation efficiencies, interactions with the ribosome or ability to suppress stop codons^10,12,35^. We show that within the *tRNA-Phe* family, the function of six out of seven phenylalanine tRNA genes have negligible adverse effects as single deletions in mice. The exception is *tRNAPhe-1-1* which appears to have a more important role and its loss leads to neurological defects or in combination with other *tRNA-Phe* deletions shifts the tipping point for lethality closer (Fig. 7g). The fact that such a defined tipping point exists, illustrates that a critical number of *tRNA-Phe* molecules is required for life and that cellular function declines precipitously beyond that point. This is reminiscent of mitochondrial disease mutation heteroplasmy, where cells can usually tolerate a high proportion of a pathogenic mutation and typically lack a phenotype until a critical threshold is exceeded and disease ensues^36^.

In summary, we describe the first systematic examination of the importance of isodecoder genes within tRNA families in mammals. Our discoveries show that these tRNA families are required to maintain functional levels of tRNAs, however, loss of single tRNA genes within a family cannot always be compensated by other isodecoders, resulting in perturbed translation of specific mRNAs. Our experimental findings and those of others^17,26^, combined with GWAS associations (Fig. 1c), and the fact that tRNA copy number has been shown to vary between humans^37^, suggest that tRNA variations may be an unrecognised source of human diversity and contribute to disease.

## Methods

### Animals and housing

Heterozygous *tRNA-Phe* gene deleted transgenic mice on a C57BL/6N background were generated by the Monash Genome Modification Platform (MGMP) and the Australian Phenomics Network (APN). Male age- and littermate-matched wild-type (WT) and homozygous mutant mice were housed in standard cages (45 cm × 29 cm × 12 cm) under a 12-h light/dark schedule (lights on 7 a.m. to 7 p.m.) in controlled environmental conditions of 22 ± 2 °C and 50 ± 10% relative humidity. Age and genotype-matched mice from different litters were used for all experiments. Normal chow diet (NCD) and water were provided ad libitum. The feed was provided by Rat & Mouse Chow, Specialty Feeds, Glen Forrest, Western Australia. The study was approved by the Animal Ethics Committee of the UWA and performed in accordance with Principles of Laboratory Care (NHMRC Australian code for the care and use of animals for scientific purposes, 8th Edition 2013).

### tRNA sequence analysis

Mature high confidence tRNA gene sets for six mammalian, two plant, two yeast and one representative of worm, fly, fish, bird and trypanosome species were obtained from GtRNAdb^38,39^. Downloaded annotations included anticodon, size, quality score, genomic location and gene sequence. For comparison of the mammalian chromosomal gene distribution, genomic coordinates were extracted and summarised into standard chromosome names. For the mammalian species, *tRNA-Phe-GAA* sequences were extracted and aligned with ClustalW^40^ to determine conservation of *tRNA-Phe* gene sequences. To investigate the potential for tRNA genes to harbour disease related sequence variations, GWAS results were obtained from GWAS Central (www.gwascentral.org^41^). Results were filtered to up- and down-stream windows of 50, 100, 250, 500 and 1000 bp of human *tRNA-Phe-GAA* genes, and hits were considered significant if *p* < 0.05. We excluded hits surrounding human *tRNA-Phe-GAA-1-4* and *tRNA-Phe-GAA-1-5* genes due to their close proximity to Pol II genes. Potential disease associations were summarised into categories.

### ChIP-Seq

ChIP-Seq information was downloaded from ChIP-Atlas peak browser annotations of the mouse reference set (mm10) for RNA polymerase antigens POLR3A, POLR3D, POLR3GL and RNA polymerase III^20^. Peak scores corresponding to *tRNA-Phe* genes were averaged to calculate generalised expression levels of each *tRNA-Phe* gene as a proxy.

### Blood analyses

Differential cell counts were performed on tail vein blood using a Hemavet HV950FS blood analyser (Drew Scientific, Waterbury, CT), calibrated using the Multi-Trol Control Mouse (ELITech Group) standard.

### Tissue homogenate preparation

3 mm × 3 mm tissue pieces (liver or brain) were homogenised in 200 μl of cell extraction buffer (CEB; 100 mM Tris, 2 mM Na_3_VO_4,_ 100 mM NaCl, 1% Triton X-100, 1 mM EDTA, 10% glycerol, 1 mM EGTA, 0.1% SDS, 1 mM NaF, 0.5% deoxycholate, 20 mM Na_4_P_2_O_7_), pH 7.4, containing PhosSTOP Phosphatase Inhibitor Cocktail (Roche) and EDTA-free Complete protease inhibitor cocktail (Roche). The homogenates were prepared using a bead beater and the homogenates were centrifuged at 9000×*g* for 5 min at 4 °C. The previous steps were repeated until a clear tissue homogenate was produced. The tissue homogenate protein concentration was quantified using the bicinchoninic acid (BCA) assay using BSA as a standard.

### Whole-genome sequencing

Total brain DNA was isolated from three control and three *tRNA-Phe-1-1*^*-/-*^ mice and whole-genome sequencing (WGS) was performed using the Illumina NovaSeq platform, according to the Illumina WGS protocol at the AGRF. The Illumina DRAGEN germline alignment and variant calling workflow (v3.9.3) was used to align reads to the reference genome (GRCm39, GCA_000001635.9), remove duplicates and perform variant calling and genotyping based on a diploid model. Default parameters were used in the analysis. The reference genome was acquired from Ensembl (v105) (https://ftp.ensembl.org/pub/release-105/fasta/mus_musculus/). Off target mutations that could be potentially introduced by Cas9 were not detected in any of the 323 potential off-targets predicted by Cas-OFFinder^42^ (allowing up to five mismatches, ±50 bp either side) (Supplementary Data 1).

### RNA isolation, northern blotting

The tissues were snap frozen and stored at −80^o^C prior to RNA isolation and RNA was isolated using the miRNeasy Mini kit (Qiagen) incorporating an on-column RNase-free DNase digestion to remove all DNA. RNA (2–5 µg) was resolved on 1.2% agarose formaldehyde gels, then transferred to 0.45 µm Hybond-N^+^ nitrocellulose membrane (GE Lifesciences) and hybridised with biotinylated oligonucleotide probes specific to nuclear tRNAs and 18 S rRNA (probe sequences are listed in Supplementary Data 4). Hybridisations were carried out overnight at 50 °C in 5x SSC, 20 mM Na_2_HPO_4_, 7% SDS and 100 µg.ml^−1^ heparin, followed by washing. The signal was detected using streptavidin-linked infrared-labelled antibody (diluted 1: 10,000 in 3x SSC, 5% SDS, 25 mM Na_2_HPO_4_, pH 7.5) by the Odyssey Infrared Imaging System (LI-COR Biosciences).

### RNA-seq

RNA sequencing was performed on total brain and liver RNA isolated from at least three WT and three *tRNA-Phe* mutant mice. Sequencing was performed using the Illumina NovaSeq platform, according to the Illumina RNA-Seq protocol and as we have done previously^43^. Sequenced reads were trimmed with Trim Galore^44^ (0.6.4_dev) using cutadapt^45^ (1.18) (with parameters:–paired–fastqc–illumina, corresponding to cutadapt parameters: -e 0.1 -q 20 -O 1 -a CTGTCTCTTATA). Trimmed reads were aligned to the mouse genome downloaded from GENCODE (ftp://ftp.ebi.ac.uk/pub/databases/gencode/Gencode_mouse/release_M24/gencode.vM24.primary_assembly.annotation.gtf.gz on 20 April, 2020) (GRCm38.p6, primary assembly) masked for nuclear mitochondrial sequences with the vM24 GENCODE mouse gene annotation (ftp://ftp.ebi.ac.uk/pub/databases/gencode/Gencode_mouse/release_M24/gencode.vM24.primary_assembly.annotation.gtf.gz on 20 April, 2020) with STAR^46^ (v2.7.8a) (–quantMode TranscriptomeSAM) with custom mitochondrial annotations. The transcriptome alignments produced by STAR were quantified with Salmon^47^ (-l ISR–seqBias–gcBias) against a transcriptome fasta produced from the GENCODE gene annotation and genome sequence with gffread^48^. The output files were imported into R (https://www.R-project.org) with tximport^49^ and tRNA knockouts were analysed for differential expression against wild types with DESeq2^50^, per tissue.

### Histology

Fresh livers were fixed with 10% neutral buffered formalin for 24 h, washed in phosphate-buffered saline (PBS) for 24 h and stored in 70% ethanol before processing. Livers were embedded in paraffin, cut in 5 µm sections using a microtome and transferred to positively charged slides. Slides were heated for 2 h at 60 °C and treated with xylene, xylene and ethanol (1:1) and decreasing concentrations of ethanol (100%, 95%, 80%, 60%), washed and hydrated in distilled water. Hematoxylin and eosin (H&E) staining was performed as described before^51^. Coverslips were attached using DPX mounting media (Scharlau) and images were acquired using a Nikon Ti Eclipse inverted microscope using a Nikon ×10 or ×20 objective.

Mice were perfused with 0.9% saline, followed by 30–50 ml of 4% paraformaldehyde, brains were extracted and stored at 4 °C, in 4% paraformaldehyde for 24 h, followed by 30% sucrose PBS solution for several days, and finally in 30% sucrose PBS solution containing 0.01% sodium azide. The right brain hemisphere was cut in the sagittal plane on a cryostat at 40 µm. Sections were either mounted on positively charged slides and stored at −80 °C until staining or stored as free-floating sections in PBS containing 0.01% sodium azide at 4 °C prior to immunohistochemistry.

Slides were equilibrated for one hour at room temperature prior to staining. Slides were stained with toluidine blue, followed by three 100% ethanol washes, and two xylene washes. Coverslips were attached with DPX mounting media (Scharlau), and images were acquired using a Nikon Ti Eclipse inverted microscope using a Nikon ×4 and ×10 objective. Free-floating sections selected for immunostaining were washed for 5 min in PBS, 15 min in 0.2% PBST, one hour in blocking buffer (10% donkey serum), and were subsequently incubated overnight at 4 °C in anti-calbindin D-28K antibody (C2724, Sigma-Aldrich) diluted 1:500. The following day sections were washed in PBS, incubated for three hours in donkey anti-rabbit Alexa Fluor 555 secondary antibody (A-31572, Thermo Fisher) diluted 1:600, stained with 1 µg/ml Hoechst 33342 (H3570, ThermoFisher), washed with PBS and mounted on positively charged slides, with fluromountG mounting medium.

The linear density of calbindin-positive Purkinje cells was analysed per lobe in the vermis and hemisphere of the cerebellum in wild-type and *tRNA-Phe1-1* knockout mice. Specifically, the number of Purkinje cells counted in each lobe was divided by the length of the Purkinje cell monolayer (µm), multiplied by 100, to give the number of Purkinje cells per 100 µm. Three sagittal sections from the vermis and three sagittal sections from the hemisphere were analysed for each mouse. Counting and measurements were carried out using the Nikon NIS-Elements software, statistical analysis was done using the Student’s *t* test.

### Behavioural analyses

All behavioural tests were carried out on ten-week old male and female mice. Prior to each experiment mice were taken to the testing room in their home cages and given 10 min to habituate. All tests were carried out in the ‘light’ phase of the light-dark cycle. To test general activity and locomotor ability mice were placed into a 36 × 26 cm box, under a constant light source. The floor of the box was divided into a 4 × 4 square grid, which was used to track their movement throughout the box. The number of boxes crossed by the mice over a 10 min period was measured, as well as the time mice spent in the inside boxes and rearing behaviour.

The mouse visual response to moving stimuli was investigated using an optokinetic drum fitted with a moving stimulus pattern of 1 cm wide black and white stripes. The drum consisted of a motorised plastic cylinder of 24 cm diameter, which rotated around a 13 cm diameter central pedestal at one revolution per minute. Mice were placed in a transparent cylindrical container on the central pedestal. Following a 2-min acclimatisation period, the mice were filmed while the drum was rotating clockwise for 2 min and then anticlockwise for 2 min, with a 1-min rest period in between. The number of tracking movements made by the mice was measured offline from video-recordings. In the tail suspension analysis mice were suspended 20 cm above a surface for 5 min. Light weight plastic tubes (5 cm long) were placed over their tails to prevent them latching onto their tails while trying to escape and the duration of escape-oriented behaviours was measured. To assess fine motor coordination and balance, mice were tested on an elevated balance beam apparatus joining two platforms. The start platform was open and placed under a light source which acted as an aversive stimulus, while the end platform was enclosed and nesting material from the home cage was added to entice the mice to traverse the beam. Mice were tested on the beam on three consecutive days, with two trials per day separated by a 10-min rest period in the home cage. The time taken to cross the beam was recorded each day as an average of the two trials.

### Analysis of mouse movement

Video recording of each subject was analysed via machine learning as implemented in DeepLabCut (v 2.2.1, http://www.mackenziemathislab.org/deeplabcut) to analyse movement^52^. Three body parts were tracked (snout, tail-base and tail-tip) and uncropped frames were extracted using the naive *k*-means clustering algorithm with the squared Euclidean distance metric from which a training dataset was created using imgaug augmenter and the network was trained with shuffle = 1 and 30,000 max iterations. Subject videos were analysed using the trained model using the arima filter method. Filtered coordinate points in pixels were then used to determine total journey length by determining the distance between coordinate points for each individual body part and scaled to real dimensions.

### Nuclei isolation and chromatin transposition

Nuclei were isolated by dissociating 20 mg of fresh brain or liver tissue in 400 µl of lysis buffer (1 M Tris-HCl, 1 M MgCl_2_, 1 M NaCl, 10% Nonidet-P40) by passing through a polished silanized Pasteur pipette. Samples were left to incubate at 4 °C for 5 min before repeated dissociation. Following an additional 5 min incubation, samples were treated with 2.5 ml of Wash buffer (1x PBS, 1% BSA), dissociated and filtered through a 30 µm cell strainer. Samples were centrifuged at 500×*g* for 5 min at 4 °C, with the remaining pellet resuspended in 110 µl wash buffer. To estimate nuclei recovery efficiency, 10 µl of nuclei solution was combined with 10 µl of DAPI solution (1:5000 in PBS) and loaded on a hematocytometer. The remaining nuclei suspension was treated with 180 µl of 1.8 M sucrose solution (2 M sucrose cushion solution (S9308-475ML), Nuclei PURE Sucrose Cushion Buffer (S9058-120ML)) and homogenised. The 280 µl of nuclei suspension was gently layered onto 1 ml of 1.3 M sucrose solution (S9308-475ML, Nuclei PURE Sucrose Cushion Buffer (S9058-120ML) and centrifuged at 3000×*g* for 10 min. Nuclei pellets were suspended in 100 µl of wash buffer.

Assays for transposase-accessible chromatin using sequencing (ATAC-Seq) were performed on nuclei samples containing 50,000 nuclei. Isolated nuclei were centrifuged at 500×*g* for 10 min at 4 °C. The pellet was suspended in transposition reaction mix (Illumina Tagment DNA TDE1 Enzyme and Buffer Kit) and incubated at 37 °C for 30 min on a thermomixer (Eppendorf) at 1000 rpm. DNA was then isolated with Zymo DNA Clean & Concentrator-5 Kit (Integrated Sciences) and DNA elutes were amplified by PCR with 25 µM of indexing primers. Library purification was performed by isolating amplified DNA with SPRIselect beads (Beckman Coulter Life Sciences) with an ethanol wash before suspension in nuclease-free water.

### ATAC-Seq analysis

Sequenced reads were trimmed, aligned to the mouse genome and filtered for PCR duplicates and alignment quality. Fragment length periodicity was estimated and used to segment the data into nucleosome-bound or -free states. Two analyses were performed with the nucleosome-free data: a genome-wide unbiased analysis of differential accessibility after calling peak regions and generating a consensus peakset; and a tRNA-focused analysis that looked at each tRNA gene ±50 nt with an average of >9000 cuts per tRNA gene. Each analysis counted the reads mapping to regions of interest and calculated differential changes using DESeq2^50^. Detailed methods are provided in the supplementary information.

### Gene ontology

Gene lists for gene ontology analysis were generated by extracting genes encoding mRNAs that overlapped with differentially expressed called peaks for ATAC-Seq results, significant proteomics results or significant transcriptomics results. PANTHER was used to perform statistical overrepresentation tests for biological process ontologies^53^. Resulting GO lists were summarised using REVIGO^54^, with allowed similarity set to small. REVIGO utlises an internal clustering algorithm that summarises GO terms and generates bubble-plots for visualisation. ATAC-Seq results for *tRNA-Phe-1-1* brain samples were visualised with CirGO^55^.

### Proteomics

Tissue homogenates were lysed in 10% SDS, 50 mM triethylammonium bicarbonate, 200 µg of protein was dissolved in 50 µl cell lysis buffer (0.13 M Tris HCl, 6 M guanidinium chloride, 0.1 M 2-chloroacetamide, 2 mM tris(2-carboxyethyl)phosphine) and digested using the S-trap mini columns as per the manufacturer’s instructions. Briefly, dithiothreitol was added to a final concentration of 20 mM and incubation at 95 °C for 10 min. Proteins were alkylated by adding iodoacetamide to a final concentration of 40 mM and incubating at room temperature in the dark for 30 min. Proteins were acidified with 2.5 µl of 12% phosphoric acid and diluted with 150 µl of binding buffer (90% methanol, 100 mM final Tris). Samples were added to the S-Trap Mini Spin columns (Protifi) by centrifugation at 4000×*g* for 30 s then subsequently washed three times by successively loading 400 µl of binding buffer and centrifuging at 4000×*g* for 30 s. Digestion was achieved by adding 1 µg sequencing-grade trypsin (Promega) and 125 µl of 50 mM triethylammonium bicarbonate and incubating for 1 h at 47 °C. Peptides were eluted by successively adding 80 µl of 50 mM triethylammonium bicarbonate, 80 µl of 0.2% aqueous formic acid and 80 µl of 50% acetonitrile in 0.2% formic acid with a 30 s centrifugation step at 4000×*g* between the addition of each elution buffer. The eluants were pooled, dried in a vacuum centrifuge and resuspended in 20 µl of buffer A (5% acetonitrile in 0.1% formic acid).

Samples were analysed using a Thermo Fisher Scientific Ultimate 3000 RSLC UHPLC and an Eclipse mass spectrometer (Thermo Fisher Scientific). Samples were injected on a reverse-phase PepMap 100 C18 trap column (5 µm, 100 Å, 150 µm i.d. × 5 mm) at a flowrate of 10 µl/min. After 2.7 min, the trap column was switched in-line with a Waters nanoEase M/Z Peptide CSH C18 resolving column (1.7 µm, 130 Å, 300 µm i.d. × 100 mm) and the peptides were eluted at a flowrate of 0.9 µl/min buffer A (5% acetonitrile in 0.1% formic acid) and buffer B (80% acetonitrile in 0.1% formic acid) as the mobile phases. The gradient consisted of: 5–24% B for 0–22 min, 24–40% B from 22 to 35 min, 40–95% B from 35 to 39 min, followed by a wash, a return of 8% buffer B and equilibration prior to the next injection. The mass spectra were obtained in DIA mode with an MS1 resolution of 60,000, automatic gain control target at 200%, maximum injection time at 40 ms and scan range from 350 to 1200 m/z. DIA spectra were recorded at resolution 15,000 and an automatic gain control target of 800%. The 70 isolation windows were 10 m/z each from mass 399.9319-1101.2502.

Data analysis was performed with Spectronaut version 14 (14.10.201222.47784) using direct DIA analysis and default settings^56^. Briefly, spectra were searched against the *Mus musculus* proteome database from UniProt (Protoeme ID UP000000589, downloaded 14/04/2020) with carbamidomethylation set as a fixed modification and methionine oxidation and N-terminal acetylation as variable with 1% false discovery rate cut-offs at the peptide spectral match, peptide and protein group levels. Quantitation was performed at the MS2 level with Q-value data filtering and cross run normalisation with Q-complete row selection. GO term analysis was performed using annotations sourced from the Mus musculus GO annotation file from the Gene Ontology Consortium (generated 25/02/2021).

A secondary analysis was performed in Spectronaut version 15 (15.0.210615.50606) to search for substituted peptides. In this analysis, a database was constructed by taking the detected proteins from the direct DIA analysis and annotating with protein sequences from UniProt (The UniProt Consortium 2021, accessed 11/01/2021) to search for specific amino acid substitution events. Each position containing a phenylalanine was substituted for each of the 20 the amino acids, resulting in a library containing the original protein sequences in addition to multiple substituted sequences for each phenylalanine position. Settings were identical to the previous run other than the introduction of the more stringent data filters in Spectronaut 15, such as the precursor posterior error probability cut-off filter, left at default settings. Peptide identifications and abundances were exported for further processing in Python 3.8. Briefly, substituted peptides were identified by screening all identified peptides against an in silico digest of the original UniProt mouse protein FASTA file using Protein Digestion Simulator (https://omics.pnl.gov/software/protein-digestion-simulator). Peptide intensities of the substituted and non-substituted peptides were added across all modifications of a single peptide (carbamidomethylation, methionine oxidation and N-acetylation) and intensities were averaged across biological replicates and the means compared using the Student’s *t* test (two-way). Substitution candidates were screened to remove peptides with identical masses (phenylalanine to oxidised methionine or phenylalanine to valine plus three oxidised methionine residues).

### Amino acid usage

Amino acid usage was calculated by taking the ratio of amino acid occurrence out of total number of amino acids for each detected protein, using the largest annotated mouse protein sequence in each case, downloaded from UniProt (The UniProt Consortium 2021, accessed 11/01/2021). To determine if there were significant differences between the amino acid content of proteins that were upregulated versus downregulated in the *Phe-1-1*^*-/-*^ knockout mice, the mean scores for each amino acid was calculated and significance was determined using a two-way Student’s *t* test.

### Immunoblotting

Specific proteins were detected using a Calretinin rabbit monoclonal antibody (ab92341) Abcam, diluted 1:500; a Calbidin-D-28K rabbit polyclonal antibody (C2724) Sigma, diluted 1:500; or a beta actin mouse monoclonal antibody (ab6276) Abcam, diluted 1:1000. IRDye 800CW goat anti-rabbit immunoglobulin G (IgG) or IRDye 680LT goat anti-mouse IgG (LI-COR Biosciences) secondary antibodies were used and the immunoblots were visualised using an Odyssey infrared imaging system (LI-COR Biosciences). Protein densitometry was determined using Image studio lite Software.

### Ribosome profiling

Ribosome profiling was performed on brain homogenates from three WT and three *tRNA-Phe* mutant mice. Brain tissue (20 mg) was homogenised under liquid nitrogen in 1 ml lysis buffer (10 mM Tris-HCl, pH 8.0, 150 mM NaCl, 5 mM MgCl_2_, 1% NP40, 10 mM DTT, 500 U/mL RNAsin, 40 mM VRC, 1% deoxycholate) and ribo-profiling was performed according to Ingolia et al^57^. Sequencing was performed using the Illumina NovaSeq platform, according to the Illumina small RNA-Seq protocol and as we have done previously^58^. TrimGalore v0.6.5^44^ using cutadapt v1.18^45^ was used in paired in mode to remove Illumina adaptor sequences. Trimmed reads were pre-aligned to the mouse reference non-coding transcripts obtained from GENCODE (gencode.vM29.ncRNA_transcripts.fa) with bowtie2 v2.4.5 using default parameters to remove non-coding transcripts. The remaining transcripts were mapped to the mouse reference genome obtained from Ensembl (Mus_musculus.GRCm39.dna_sm.primary_assembly.fa, Mus_musculus.GRCm39.106.gff3) using STAR v2.7.8a^46^ (–alignEndsType Extend5pOfRead1 –outFilterMatchNminOverLread 0.9 –outFilterMunltimapNmax 3 –alignIntronMax 2500). Aligned reads were analysed for ribosome footprints using fivepseq v1.0.0^59^ with the same mouse reference gff3 file used for alignment and using cumulative scores for control vs knockout comparisons.

### Reporting summary

Further information on research design is available in the Nature Portfolio Reporting Summary linked to this article.

## Supplementary information

Supplementary Information

Peer Review File

Description of Additional Supplementary Files

Supplementary Data 1 - 4

Reporting Summary

## Data Availability

Transcriptomic data are deposited to the Gene Expression Omnibus (GEO) as GSE223662, whole-genome sequencing data are deposited in Biosamples as PRJNA777439 and the mass spectrometry proteomics data have been deposited to the ProteomeXchange (http://proteomecentral.proteomexchange.org) PXD029684 via the PRIDE partner repository. ATAC-Seq data are deposited to the Gene Expression Omnibus (GEO) as GSE200783. The scripts for the analyses are deposited in GitHub: https://github.com/ORAFLAB/Hughes_et_al_2023. Source data are provided with this paper.

## Source data

Source Data
