# Supplementary material for: Copy number variation in tRNA isodecoder genes impairs mammalian development and balanced translation
Source: Nat Commun. 2023 Apr 18;14:2210. doi: 10.1038/s41467-023-37843-9 (PMC10113395; doi:10.1038/s41467-023-37843-9)
Supplement: Supplementary file 1 — Supplementary Information [file 41467_2023_37843_MOESM1_ESM.pdf]

Supplemental Information

Copy number variation in tRNA isodecoder genes impairs mammalian development and balanced translation

Laetitia A. Hughes[1,2,3†], Danielle L. Rudler[1,2,3†], Stefan J. Siira[1,2,3], Tim McCubbin[4,11], Samuel A. Raven[1,2,3], Jasmin M. Brown[1,2,3], Judith A. Ermer[1,2,3], Jeanette Rientjes[5], Jennifer Rodger[6,7], Esteban Marcellin[2,4,8], Oliver Rackham[1,2 ,9,10,11*] and Aleksandra Filipovska[1,2,3,11,12*]

[1]Harry Perkins Institute of Medical Research and [2]ARC Centre of Excellence in Synthetic Biology, QEII Medical Centre, Nedlands, Western Australia 6009, Australia
[3]Centre for Medical Research, The University of Western Australia, QEII Medical Centre, Nedlands, Western Australia 6009, Australia
[4]Australian Institute for Bioengineering and Nanotechnology, The University of Queensland, 4072 Queensland, Australia
[5]Monash University
[6]School of Human Sciences (Physiology), The University of Western Australia, Crawley, Western Australia 6009, Australia
[7]Perron Institute for Neurological and Translational Sciences, Nedlands, Western Australia 6009, Australia
[8]Queensland Metabolomics and Proteomics (Q-MAP), The University of Queensland, 4072 Queensland, Australia
[9]School of Pharmacy and Biomedical Sciences, Curtin University, Bentley, Western Australia 6102, Australia
[10]Curtin Health Innovation Research Institute, Curtin University, Bentley, Western Australia 6102, Australia
[11]Telethon Kids Institute, Northern Entrance, Perth Children's Hospital, 15 Hospital Avenue, Nedlands, Western Australia, Australia
[12]School of Molecular Sciences, The University of Western Australia, Crawley, Western Australia 6009, Australia

[†]Co-first authors
[*]Co-Lead contacts for correspondence: oliver.rackham@curtin.edu.au and aleksandra.filipovska@uwa.edu.au

CONTENTS

Supplementary Figure 1 related to Figure 1. Tissue-specific expression of *tRNA-Phe* genes.

Supplementary Figure 2 related to Figure 2. CRISPR-Cas deletion of *tRNA-Phe* genes.

Supplementary Figure 3 related to Figure 2. Loss of *tRNA-Phe* genes leads to changes in the blood.

Supplementary Figure 4 related to Figure 3. Transcriptome-wide molecular signatures in the absence of specific *tRNA-Phe* genes.

Supplementary Figure 5 related to Figure 3. Volcano plots of transcriptomic changes in brain and liver of specific *tRNA-Phe* knockout mice.

Supplementary Figure 6 related to Figure 3. Proteome-wide molecular signatures in brain and liver in the absence of specific *tRNA-Phe* genes.

Supplementary Figure 7 related to Figure 3. Volcano plots of proteomic changes in brain and liver of specific *tRNA-Phe* knockout mice.

Supplementary Figure 8 related to Figure 4. Loss of *tRNA-Phe* genes affects behaviour.

Supplementary Figure 9 related to Figure 5. Tissue-specific expression changes of *tRNA-Phe* genes in specific *tRNA-Phe* knockout mice.

Supplementary Figure 10 related to Figure 5. Chromatin accessibility changes at *tRNA-Phe* loci in response to specific *tRNA-Phe* knockout mice.

Supplementary Figure 11 related to Figure 6. Changes in genomic arrangement in the absence of specific *tRNA-Phe* genes in liver.

Supplementary Figure 12 related to Figure 6. Gene ontologies based on ATAC-Seq changes in brain and livers from specific *tRNA-Phe* knockout mice.

Supplementary Figure 13 related to Figure 6. Reduction in the abundance of specific phenylalanine-rich proteins involved in neurotransmission was found in the brain proteomes of *tRNA-Phe-1-1* mice.

Supplementary Figure 14 related to Figure 6. Proteomic spectra of mistranslated peptides in the brains of *tRNA-Phe-1-1* knockout mice.

Supplementary Figure 15 related to Figure 6. Ribosome profiling identified stalling at phenylalanine codons in the brains of *tRNA-Phe-1-1* knockout mice.

Extended Methods

Supplementary Data 1. Off target effects in WGS data.

Supplementary Data 2. Differential expression of genes in tRNA-Phe knockout mice compared to controls.

Supplementary Data 3. Protein changes in tRNA-Phe knockout mice compared to controls.

Supplementary Data 4. Northern blotting probe sequences.

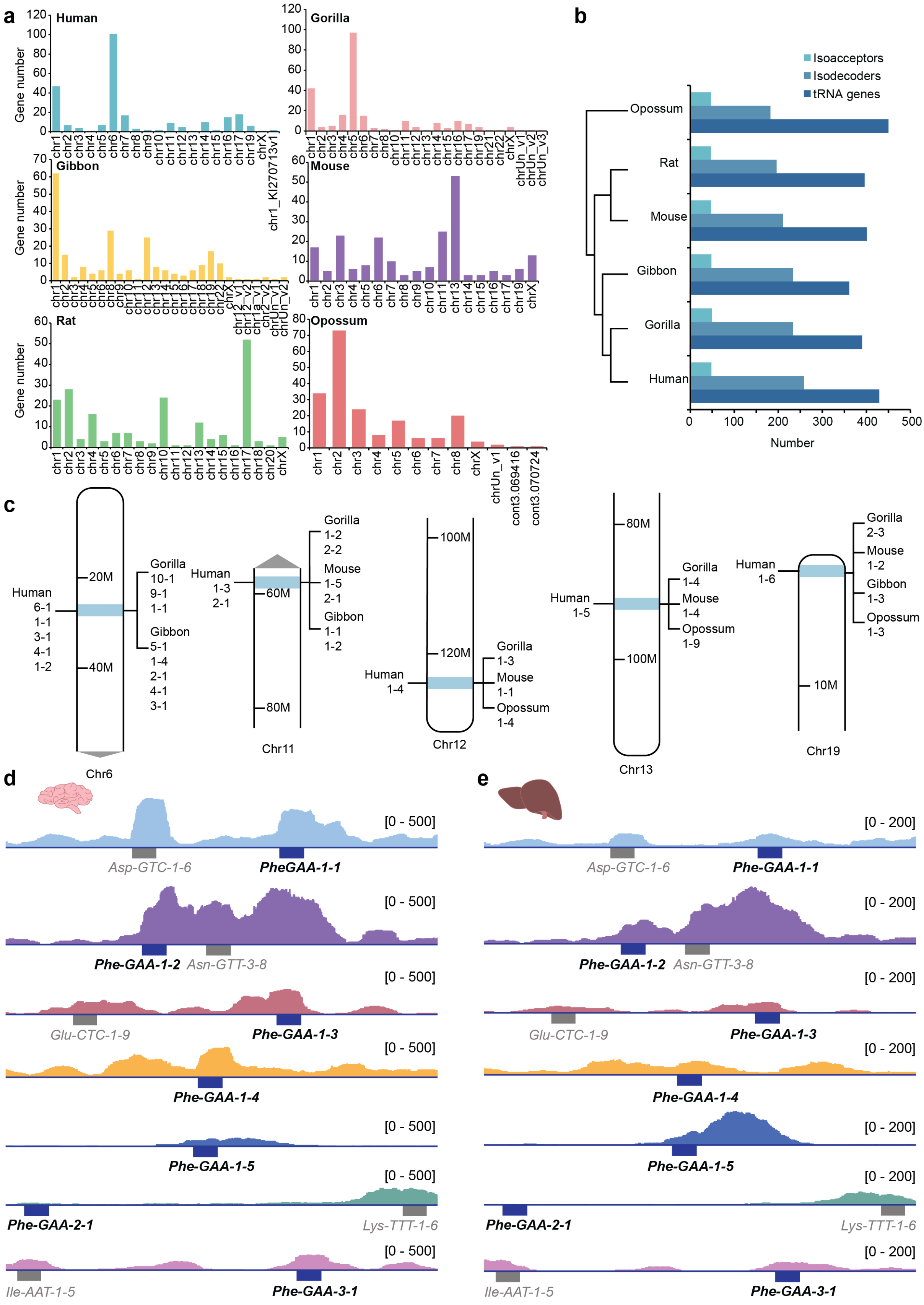
a
Human
Gorilla
Gibbon
Mouse
Rat
Opossum
Gene number
b
Isoacceptors
Isodecoders
tRNA genes
Opossum
Rat
Mouse
Gibbon
Gorilla
Human
Number
c
Chr6
Chr11
Chr12
Chr13
Chr19
d
e
Asp-GTC-1-6
PheGAA-1-1
Phe-GAA-1-1
Phe-GAA-1-2
Asn-GTT-3-8
Glu-CTC-1-9
Phe-GAA-1-3
Phe-GAA-1-4
Phe-GAA-1-5
Phe-GAA-2-1
Lys-TTT-1-6
Ile-AAT-1-5
Phe-GAA-3-1
[0 - 500]
[0 - 200]

Supplementary Figure 1 related to Figure 1. Tissue-specific expression of *tRNA-Phe* genes. **a**, Distribution of tRNA genes over chromosomes from six mammalian species, summarized into standard chromosome names. **b**, Number of total tRNA genes, isoacceptors and isodecoders in six mammals, with evolutionary relationships indicated. **c**, Syntenic regions of the *tRNA-Phe* genes between the six mammalian species was determined through pairwise alignments from Ensembl (https://asia.ensembl.org/index.html). **d-e**, Genome browser view of the mean chromatin accessibility of the seven *tRNA-Phe* genes isolated from three control mice, determined by ATAC-seq in **c**, brain and **d**, liver, showing the standard expression of each *tRNA-Phe* gene relative to each other. Peak sizes represent pileup score as calculated by Genrich with tRNA gene locations annotated beneath peak tracks from GtRNAdb (scale, 0 to 500 in brain, 0 to 200 in liver).

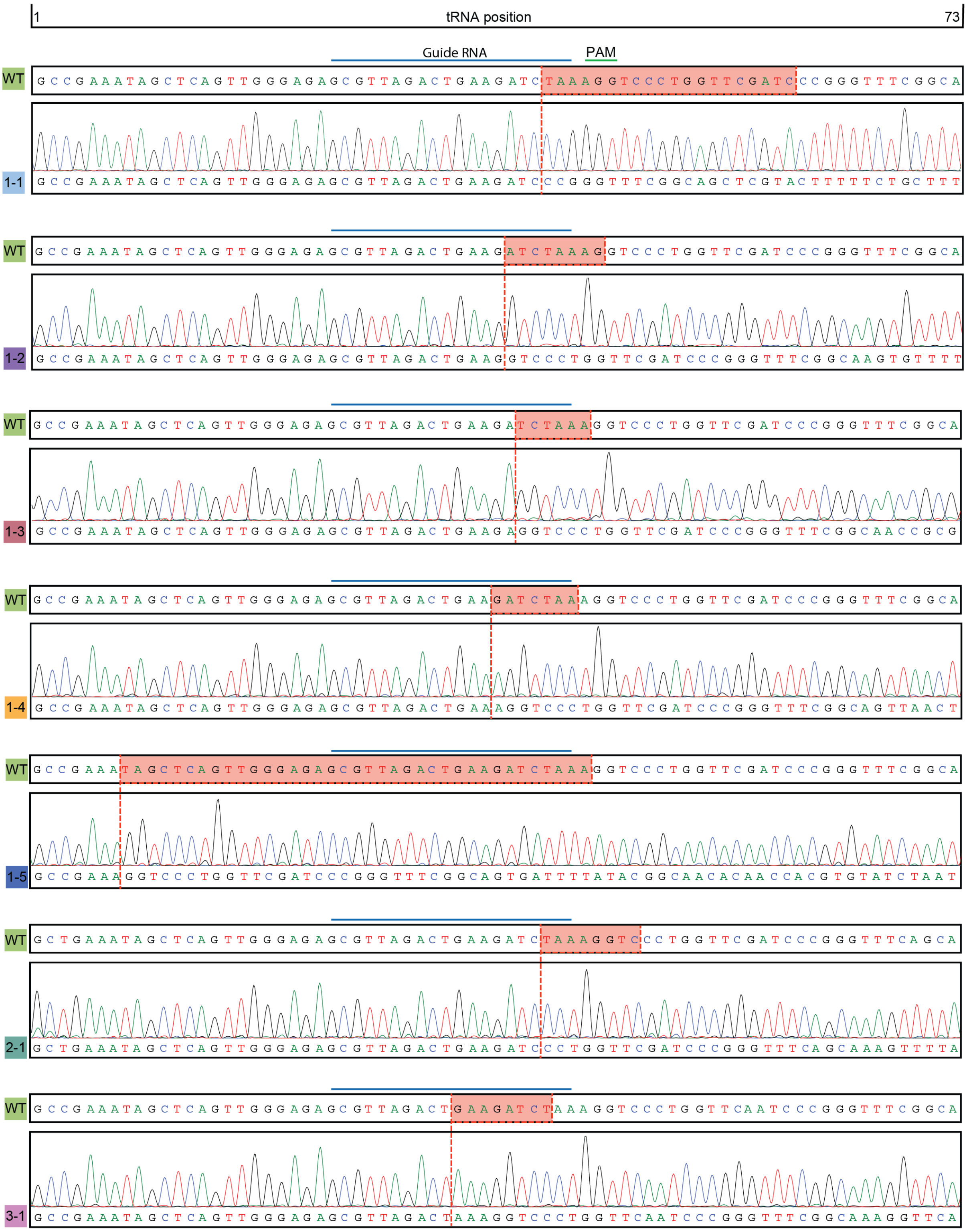

Supplementary Figure 2 related to Figure 2. CRISPR-Cas deletion of *tRNA-Phe* genes. Sanger sequencing of DNA extracted from mouse tail tips confirmed the size of deletions within each of the seven *tRNA-Phe* isodecoder genes leading to their knockout. Chromatograms

are shown for each mouse line and are compared to the wild-type sequence. Locations of the gRNA target and PAM sequence are shown above the wild-type *tRNA-Phe* sequence.

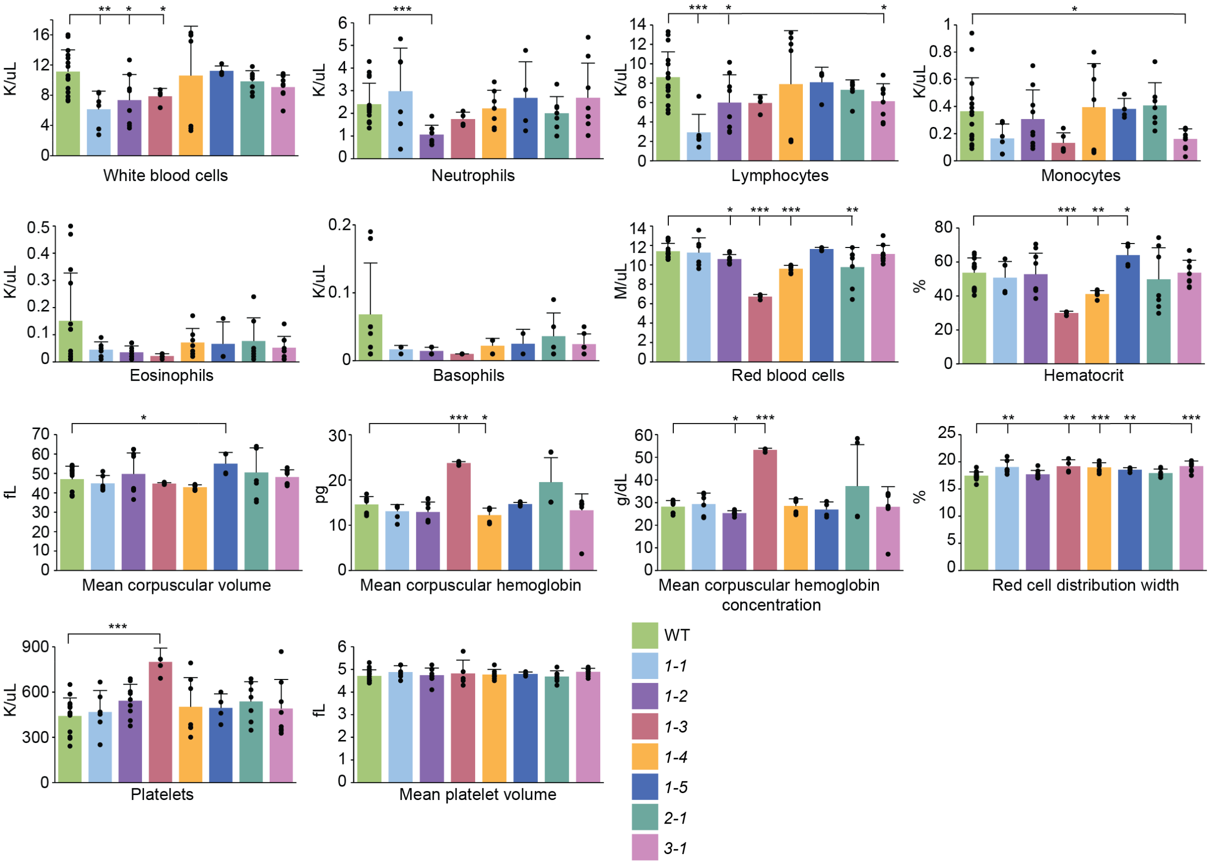

Supplementary Figure 3 related to Figure 2. Loss of *tRNA-Phe* genes lead to changes in the blood. Blood samples from 10-week-old mice were analysed and counts for each specific class of blood cell are shown. All values are means ± SD of n=3. * $p < 0.05$, ** $p < 0.01$, *** $p < 0.001$, Student's two-way *t*-test; (p= 0.0014 for white blood cells *tRNA-Phe-1-1*, p= 0.01 for white blood cells *tRNA-Phe-1-2*, p= 0.043 for white blood cells *tRNA-Phe-1-3*; p= 0.00046 for neutrophils *tRNA-Phe-1-3*; p= 0.0001 for lymphocytes *tRNA-Phe-1-1*, p= 0.036 for lymphocytes *tRNA-Phe-1-2*, p= 0.026 for lymphocytes *tRNA-Phe-3-1*; p= 0.025 for monocytes *tRNA-Phe-3-1*; p= 0.01 for red blood cells *tRNA-Phe-1-2*, p= 0.0000000011 for red blood cells *tRNA-Phe-1-3*, p= 0.0000012 for red blood cells *tRNA-Phe-1-4,* p= 0.0097 for red blood cells *tRNA-Phe-2-1*; p= 0.00005 for hematocrit *tRNA-Phe-1-3*, p= 0.0014 for hematocrit *tRNA-Phe-1-4,* p= 0.042 for hematocrit *tRNA-Phe-1-5*; p= 0.042 for MCV *tRNA-Phe-1-5*; p= 0.0000027 for MCH *tRNA-Phe-1-3*, p= 0.025 for MCH *tRNA-Phe-1-4;* p= 0.014 for MCHC *tRNA-Phe-1-2*, p= 0.0000000065 for MCHC *tRNA-Phe-1-3;* p= 0.0011 for RCDW *tRNA-Phe-1-1*, p=

0.0011 for RCDW *tRNA-Phe-1-3*, p= 0.00016 for RCDW *tRNA-Phe-1-4,* p= 0.0081 for RCDW *tRNA-Phe-1-5,* p= 0.000016 for RCDW *tRNA-Phe-3-1*; p= 0.000038 for platelets *tRNA-Phe-1-3*.

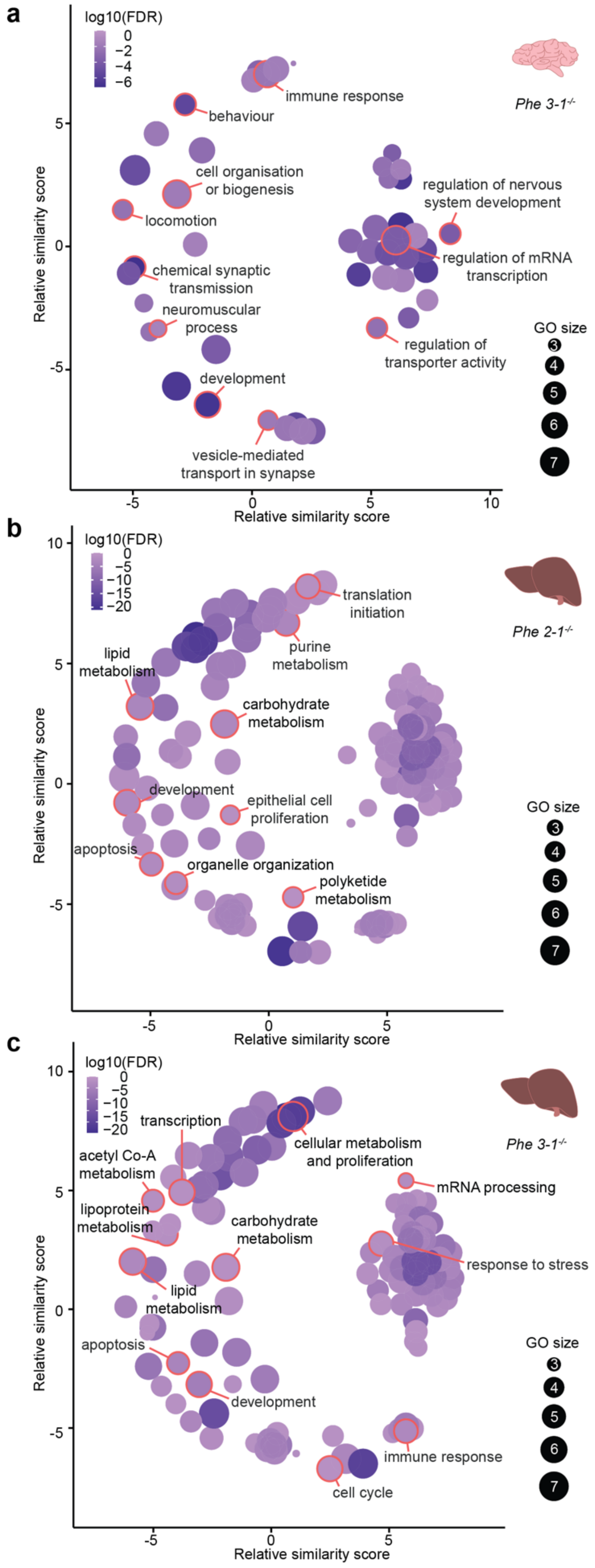

Supplementary Figure 4 related to Figure 3. Transcriptome-wide molecular signatures in the absence of specific *tRNA-Phe* genes. Differential gene expression analyses of RNA-

seq data from knockout and control mice summarised by biological process GOs using PANTHER showing changes in *Phe 3-1*$^{-/-}$ in the brain **a**, *Phe 2-1*$^{-/-}$ in the liver **b**, and *Phe 3-1*$^{-/-}$ in the liver **c**. GO results were visualized using REVIGO, where GO size represents the number of total genes in each specific ontology, and the colour scale represents the degree of significance.

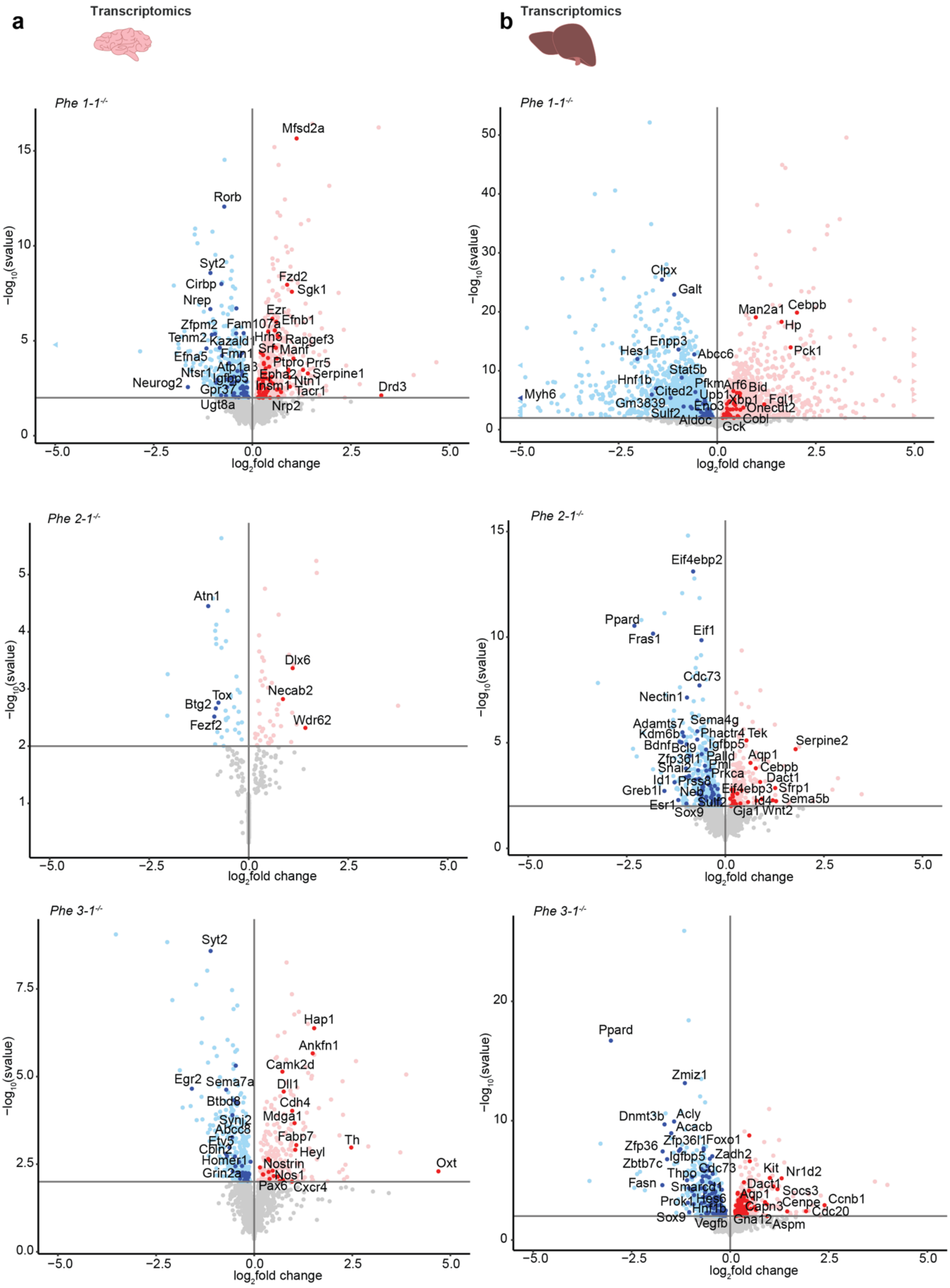

Supplementary Figure 5 related to Figure 3. Volcano plots of brain (**a**) and liver (**b**) transcriptome changes in specific *tRNA-Phe* knockout lines (n=3 per genotype). Significantly

increased genes are shown in red and significantly reduced genes are shown in blue, specific genes related to neurological and liver function that are significantly reduced or increased are shown in dark blue or dark red, respectively.

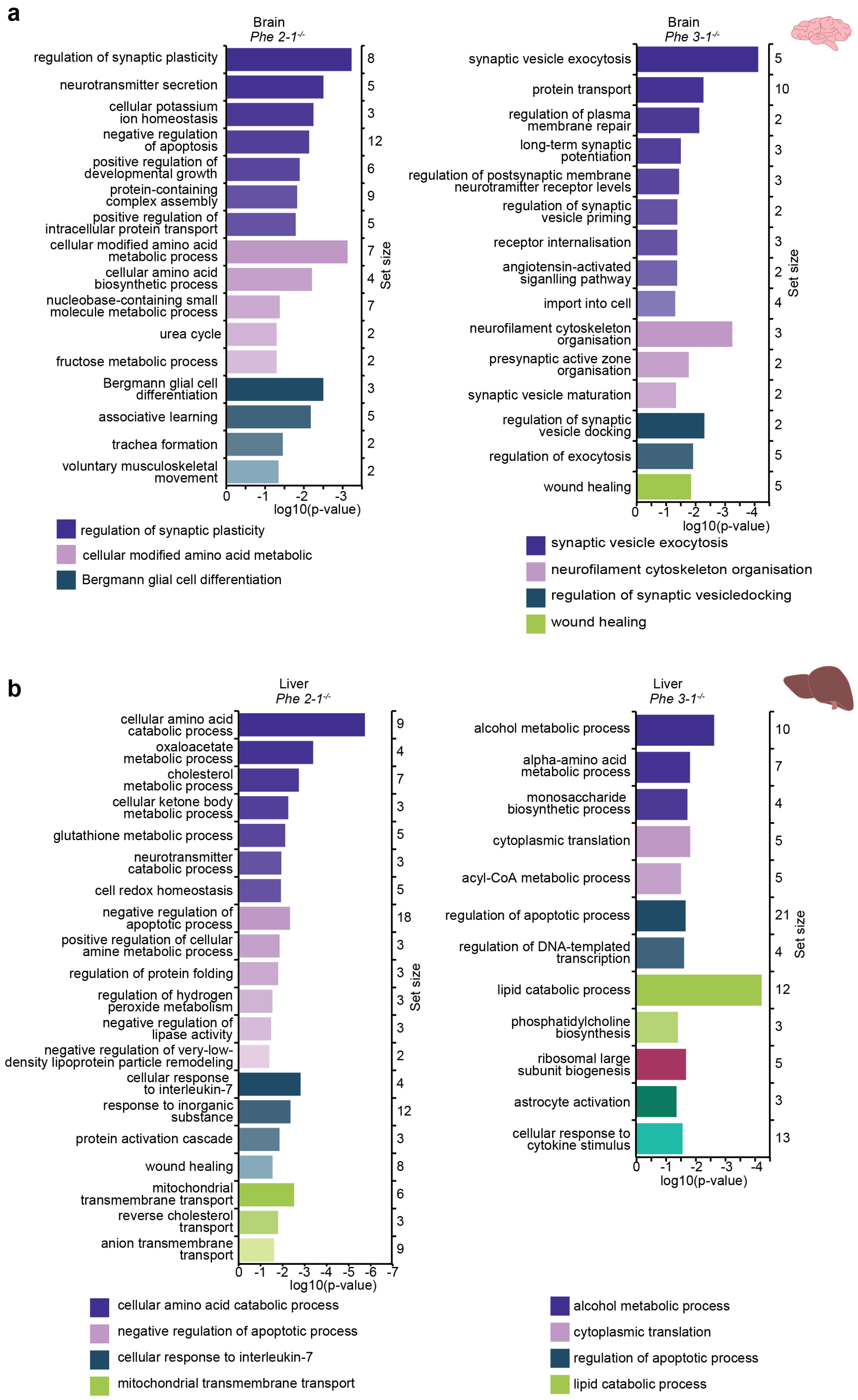

Supplementary Figure 6 related to Figure 3. Proteome-wide molecular signatures in brain and liver in the absence of specific *tRNA-Phe* genes. Proteomic analyses results from

brain and liver of *tRNA-Phe* knockout mice summarized by biological process GOs determined using PANTHER and REVIGO for **a**, *Phe 2-1*$^{-/-}$ and *Phe 3-1*$^{-/-}$ mouse knockouts in brain and **b**, *Phe 2-1*$^{-/-}$ and *Phe 3-1*$^{-/-}$ mouse knockouts in the liver compared to controls. False discovery rate (FDR) of < 0.05 was used to pick significantly enriched GO terms.

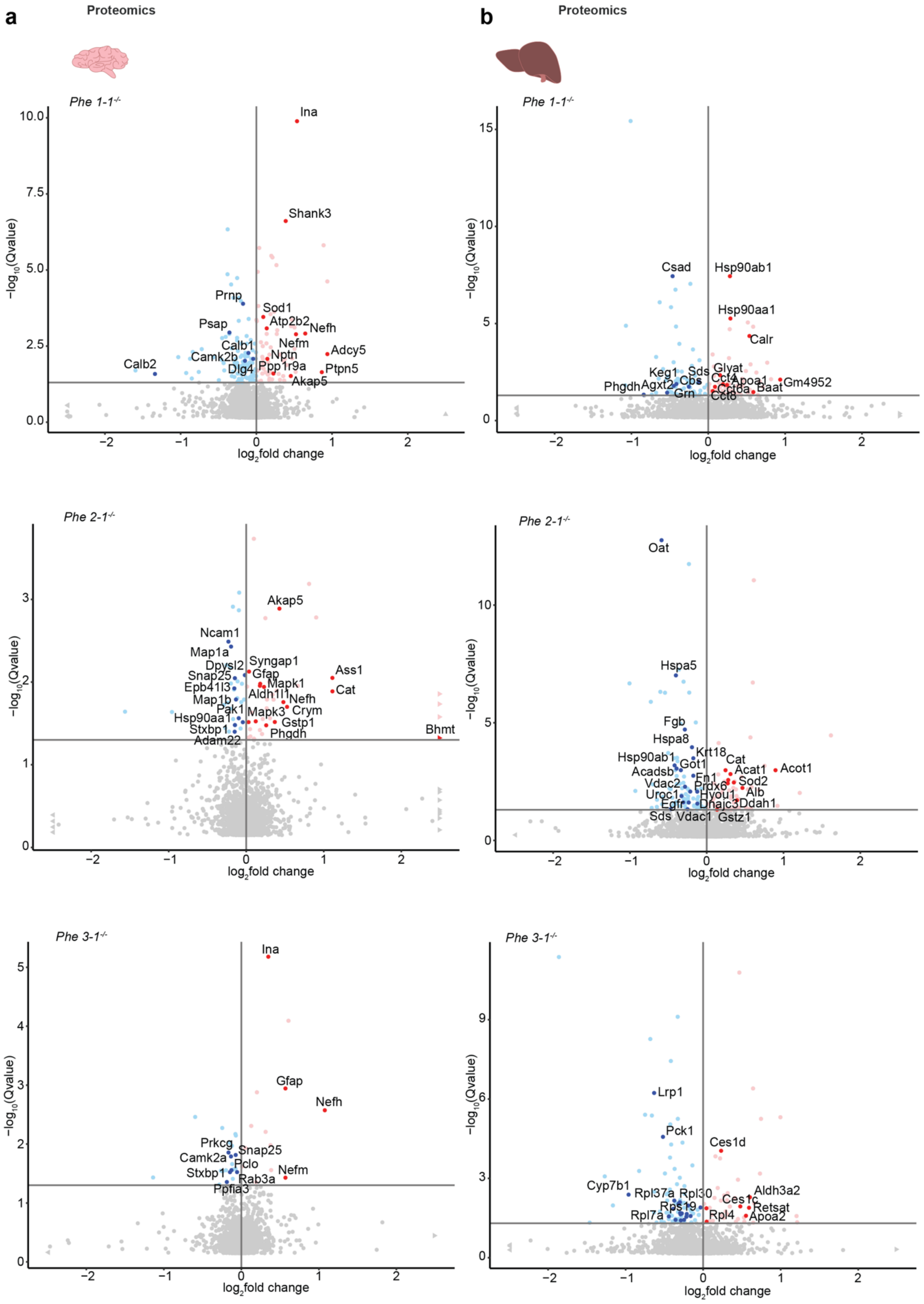

Supplementary Figure 7 related to Figure 3. Volcano plots of brain (**a**) and liver (**b**) proteome changes in specific *tRNA-Phe* knockout lines (n=5 per genotype). Significantly

increased proteins are shown in red and significantly reduced proteins are shown in blue, specific proteins related to neurological and liver function that are significantly reduced or increased are shown in dark blue or dark red, respectively.

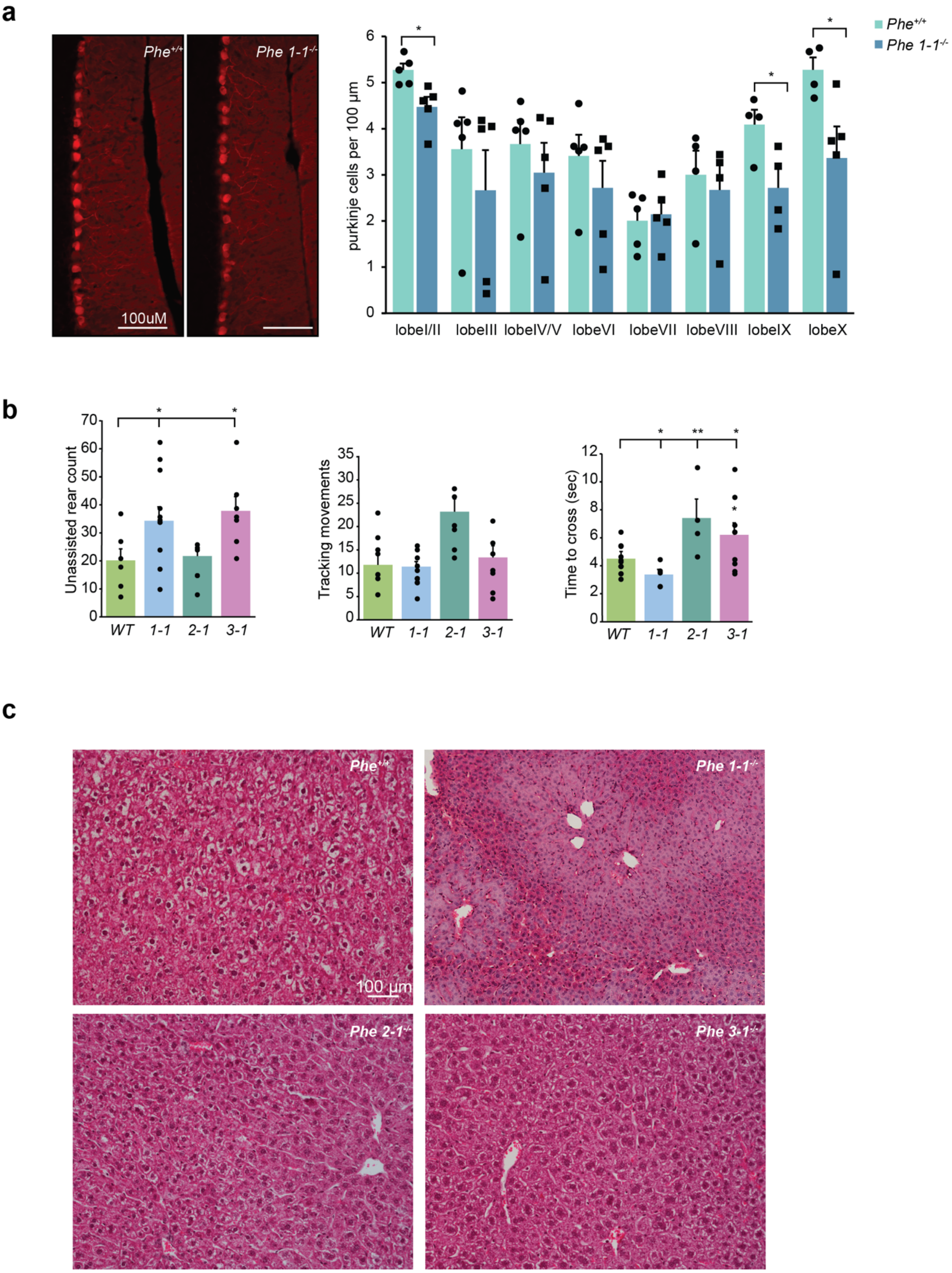

Supplementary Figure 8 related to Figure 4. Loss of *tRNA-Phe* genes affects behaviour. **a**, Immunostaining with calbindin in the vermis of the cerebellum revealed an overall decrease in calbindin positive Purkinje cells in the *tRNA-Phe-1-1*$^{-/-}$ knockout mice compared to controls

(n=5 of each genotype). All values are means ± SD of n=5 * $p < 0.05$, (p= 0.043 for lobe IX and p= 0.047 for Lobe X), two-sided Student's *t*-test. **b**, Open field behavioural assessment of *tRNA-Phe-1-1*$^{-/-}$, *tRNA-Phe-2-1*$^{-/-}$ and *tRNA-Phe-3-1*$^{-/-}$ mice compared to controls (n=7), measuring the number of rearing movements observed over a ten-minute testing period. Evaluation of tracking ability during a four-minute optokinetic drum test, measured in number of tracking movements made by the mice. Analysis of balance beam performance (p= 0.044 for *tRNA-Phe-1-1*$^{-/-}$ and p= 0.026 for *tRNA-Phe-3-1*$^{-/-}$), measured in time to cross the beam (p= 0.038 for *tRNA-Phe-1-1*$^{-/-}$, p= 0.0043 for *tRNA-Phe-2-1*$^{-/-}$ and p= 0.04 for *tRNA-Phe-3-1*$^{-/-}$) (n=7); All values are means ± SD for two-sided Student's t-test. **c**, Liver sections from 10-week-old mice cut at 5 µm, stained with hematoxylin and eosin; experiments were repeated five times with similar results.

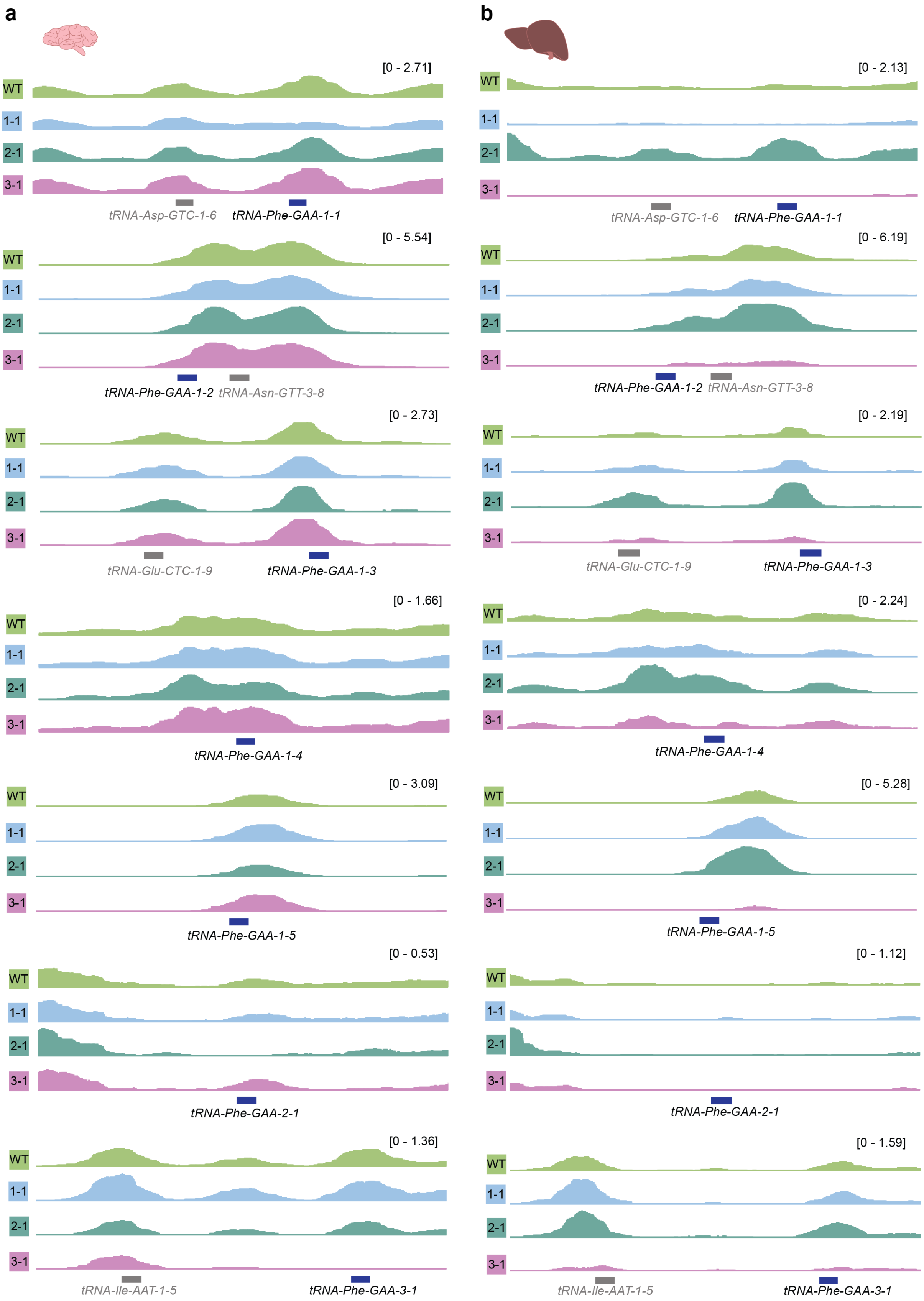
a
b
[0 - 2.71]
WT
1-1
2-1
3-1
tRNA-Asp-GTC-1-6
tRNA-Phe-GAA-1-1
[0 - 2.13]
[0 - 5.54]
tRNA-Phe-GAA-1-2
tRNA-Asn-GTT-3-8
[0 - 6.19]
[0 - 2.73]
tRNA-Glu-CTC-1-9
tRNA-Phe-GAA-1-3
[0 - 2.19]
[0 - 1.66]
tRNA-Phe-GAA-1-4
[0 - 2.24]
[0 - 3.09]
tRNA-Phe-GAA-1-5
[0 - 5.28]
[0 - 0.53]
tRNA-Phe-GAA-2-1
[0 - 1.12]
[0 - 1.36]
tRNA-Ile-AAT-1-5
tRNA-Phe-GAA-3-1
[0 - 1.59]

Supplementary Figure 9 related to Figure 5. Tissue-specific expression changes of *tRNA-Phe* genes in specific *tRNA-Phe* knockout mice. Genome browser view of the mean chromatin accessibility (normalized reads) of the seven *tRNA-Phe* genes in three control mice (WT) and three of *Phe 1-1*$^{-/-}$ (1-1), *Phe 2-1*$^{-/-}$ (2-1) and *Phe 3-1*$^{-/-}$ (3-1) knockout mice, determined by ATAC-seq in **a**, brain and **b**, liver, highlighting the changes in expression of each *tRNA-Phe* gene relative to each other. Peak sizes represent normalized reads with tRNA gene locations annotated beneath peak tracks from GtRNAdb. Scales are indicated above each set of tracks.

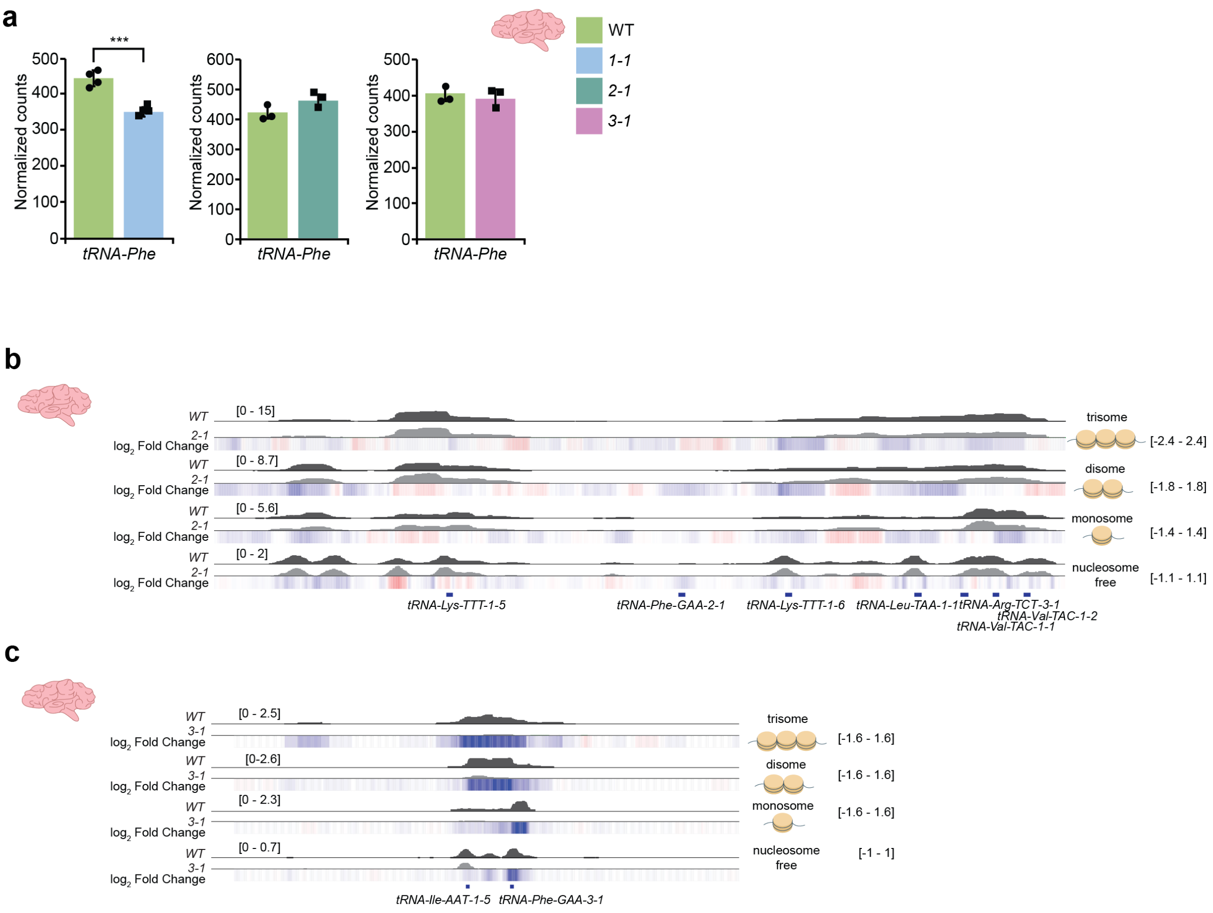

Supplementary Figure 10 related to Figure 5. Chromatin accessibility changes at *tRNA-Phe* loci in response to specific *tRNA-Phe* deletions in the brain. a, ATAC-Seq was used to identify changes in chromatin accessibility (normalised counts) of the summed up seven *tRNA-Phe* genes in control mice compared to *tRNA-Phe-1-1*$^{-/-}$, *tRNA-Phe-2-1*$^{-/-}$ and *tRNA-Phe-3-1*$^{-/-}$ knockout mice in brain. Results show the mean normalised count for each set of replicates (n=3 of each genotype) over *tRNA-Phe* gene regions (in different colours) for each of the three knockout mouse lines compared to control mice in brain; all values are means ± SD. *** $p <$ 0.01, Student's two-way *t*-test (p= 0.00082 for *tRNA-Phe-1-1*). **b**, ATAC-Seq tracks show changes in the accessibility of nucleosome-free, mono-, di- and trinucleosome-bound chromatin within the *tRNA-Phe-2-1* locus of *tRNA-Phe-2-1*$^{-/-}$ mice and, **c**, within the *tRNA-*

Phe-3-1 locus in the brain in response to loss of *tRNA-Phe-3-1* compared to controls. Coverage tracks are expressed in reads per million (RPM) and heatmap tracks show log_2 fold change.

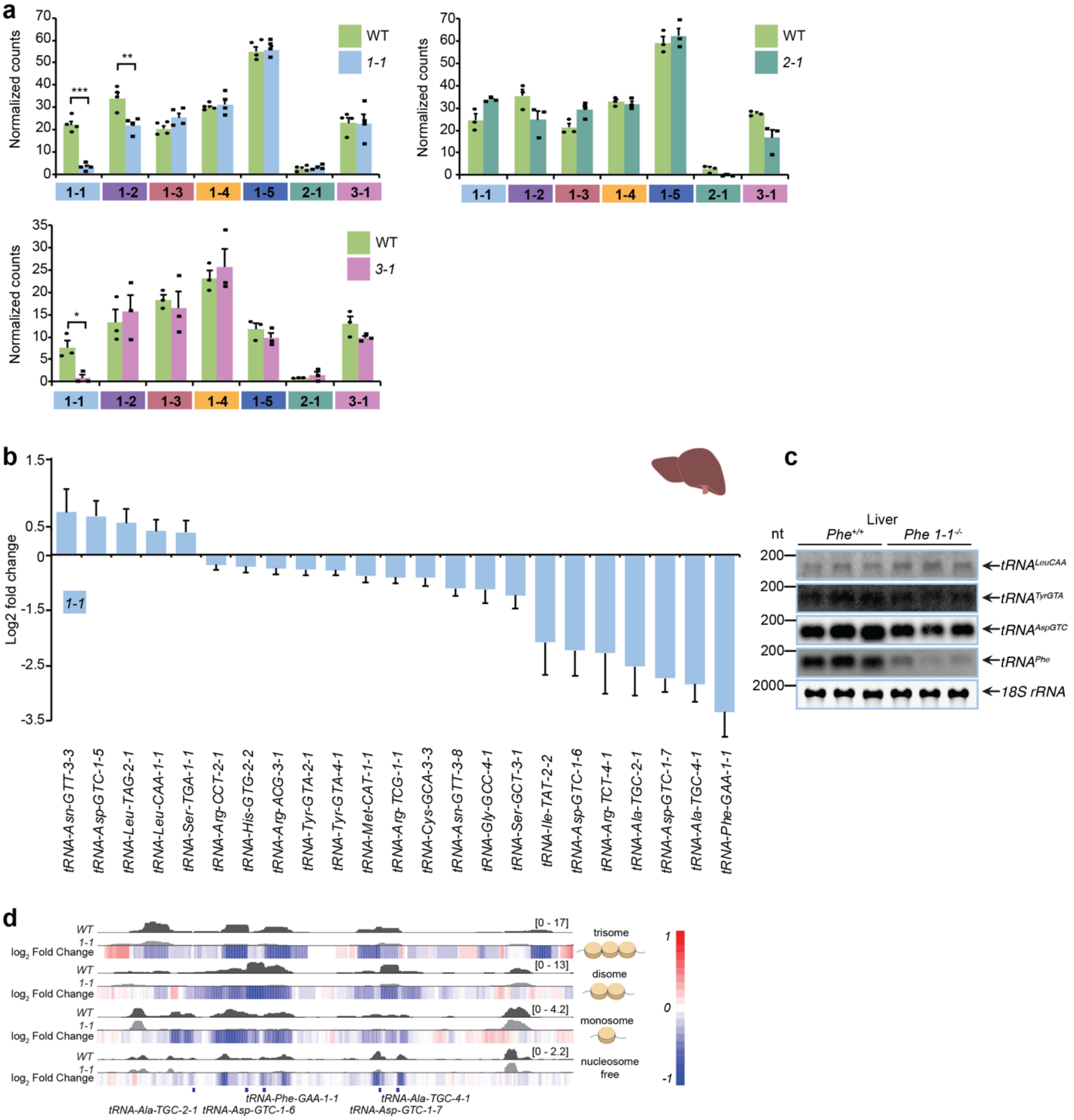

Supplementary Figure 11 related to Figure 5. Chromatin accessibility changes at *tRNA* loci in response to specific *tRNA-Phe* deletions in the liver. ATAC-seq was used to identify changes in chromatin accessibility of the seven *tRNA-Phe* genes in control mice compared to *tRNA-Phe* deletions in liver. **a**, Results show the normalized counts for each set of replicates (n=3 per mouse line) over the *tRNA-Phe* gene regions (in different colour) for each of the knockout mouse lines compared to control mice, determined using ATAC-seq. n=3; all values are means ± SEM. * $p < 0.05$, ** $p < 0.01$, *** $p < 0.001$, Student's two-way t-test (p= 0.00037

for *tRNA-Phe-1-1,* p= 0.009 for *tRNA-Phe-1-2* loci in the *tRNA-Phe-1-1*$^{-/-}$mice and p= 0.032 for *tRNA-Phe-1-12* loci in the *tRNA-Phe-3-1*$^{-/-}$). **b**, Significant changes in expression of all tRNA genes in livers of *tRNA-Phe-1-1* knockout mice compared to controls, determined using ATAC-seq, n=3, all values are means ± SD. **c**, Northern blotting of altered tRNAs in livers of *tRNA-Phe-1-1* knockout mice compared to controls (n=3) and similar results were seen with two additional independent blots. **d**, ATAC-Seq tracks show changes in the accessibility of nucleosome-free, mono-, di- and trinucleosome-bound chromatin within the *tRNA-Phe-1-1* locus in livers of *tRNA-Phe-1-1*$^{-/-}$ mice compared to controls. Coverage tracks are expressed in reads per million (RPM) and heatmap tracks show $\log_2$ fold change.

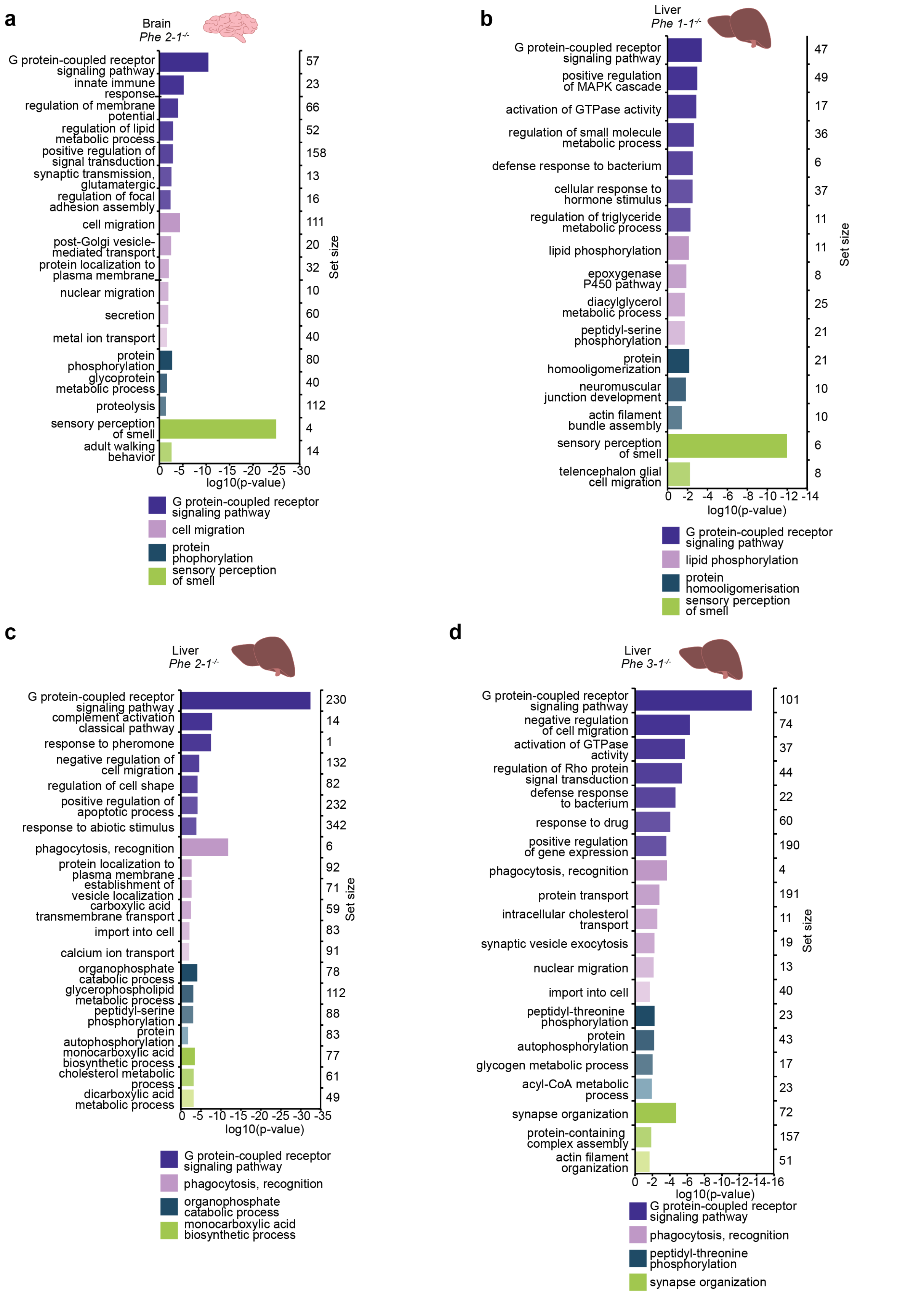

Supplementary Figure 12 related to Figure 6. Changes in genomic arrangement in the absence of specific *tRNA-Phe* genes in brain and liver. a-d, ATAC-seq results in brain and

liver show the changes in mRNA encoding genes summarised as biological process gene ontologies determined by PANTHER and visualized using REVIGO for *tRNA-Phe-2-1*$^{-/-}$ in brain **a**, and *tRNA-Phe-1-1*$^{-/-}$ in liver **b**, *tRNA-Phe-2-1*$^{-/-}$ in liver **c**, and *tRNA-Phe-3-1*$^{-/-}$ in liver **d**. False discovery rate (FDR) of < 0.05 was used to pick significantly enriched GO terms.

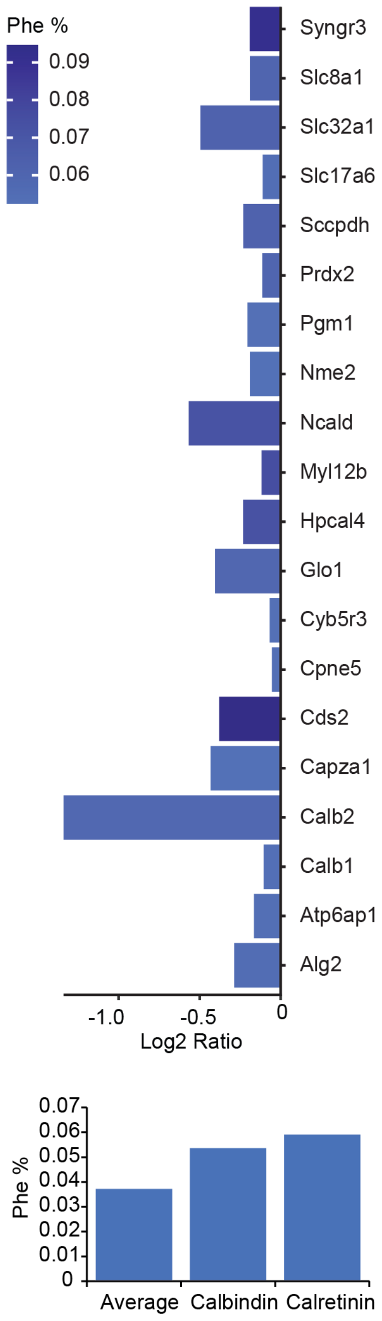

Supplementary Figure 13 related to Figure 6. Reduction in the abundance of specific phenylalanine-rich proteins involved in neurotransmission was found in the brain proteomes of *tRNA-Phe*$^{1\text{-}1}$ mice. Average log2 ratio of proteins that were decreased the most in *tRNA-Phe-1-1* knockout mice with phenylalanine percent for each one indicated by the colour scale. Phenylalanine content in calbindin and calretinin is shown relative to average phenylalanine containing proteins identified in the brain proteomes of *tRNA-Phe-1-1*$^{-/-}$ knockout mice.

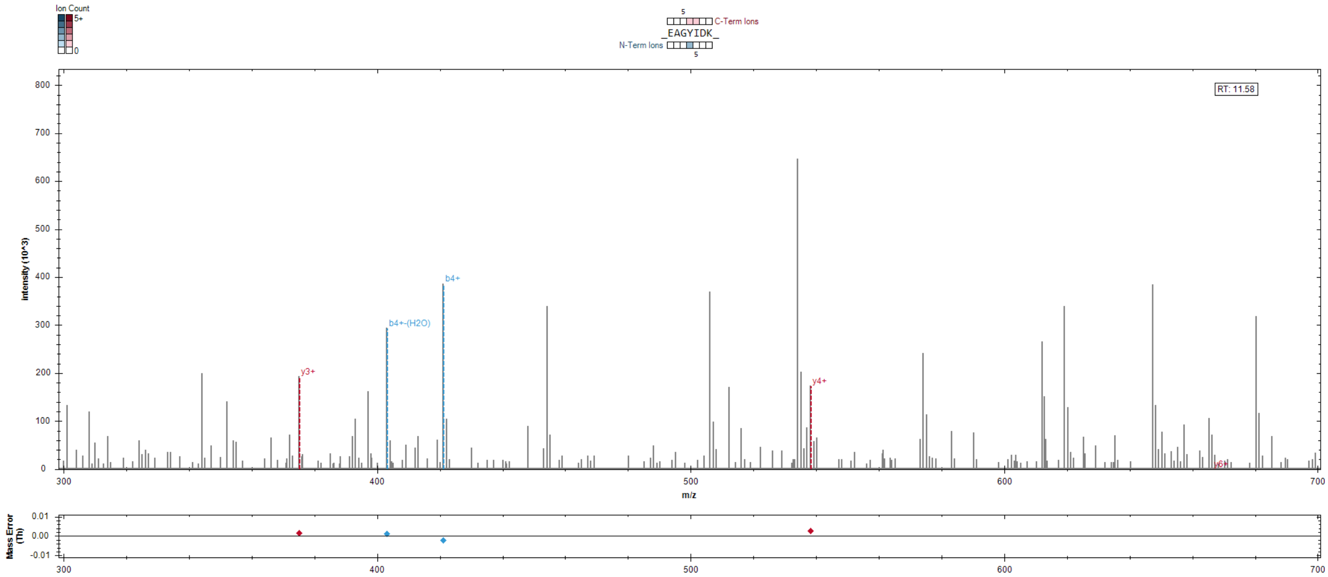
Ion Count
5+
0
C-Term Ions
EAGYIDK
N-Term Ions
RT: 11.58
Intensity (10^3)
b4+
b4+-(H2O)
y3+
y4+
m/z
Mass Error (Th)

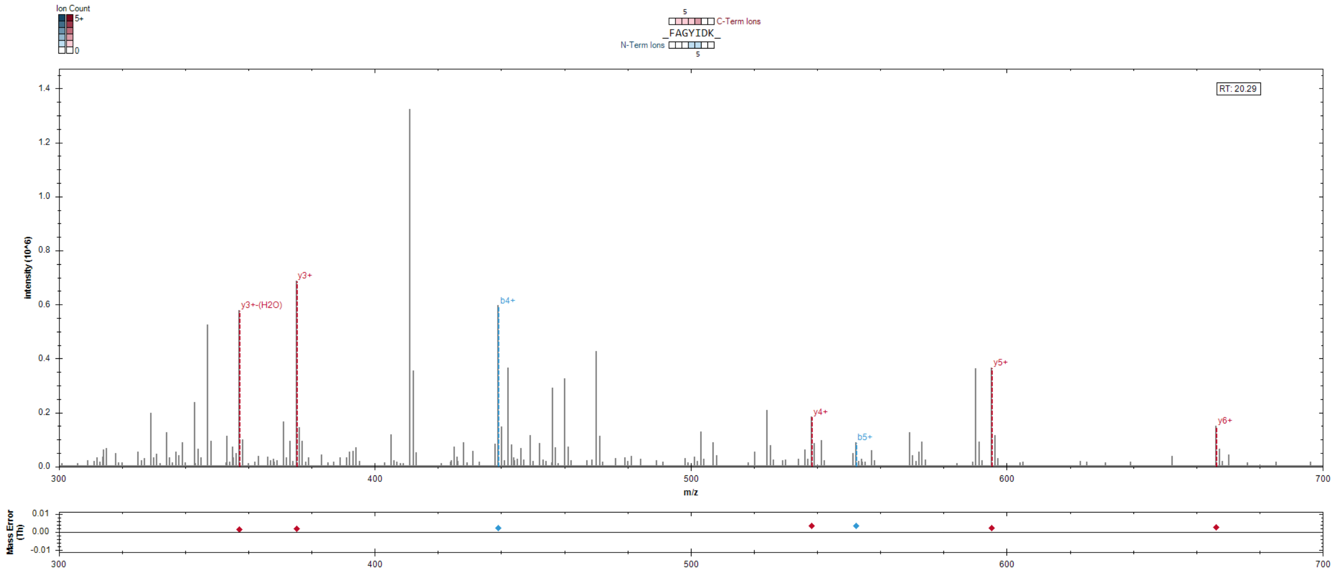
Ion Count
5+
0
C-Term Ions
FAGYIDK
N-Term Ions
RT: 20.29
Intensity (10^6)
y3+
y3+-(H2O)
b4+
y4+
b5+
y5+
y6+
m/z
Mass Error (Th)

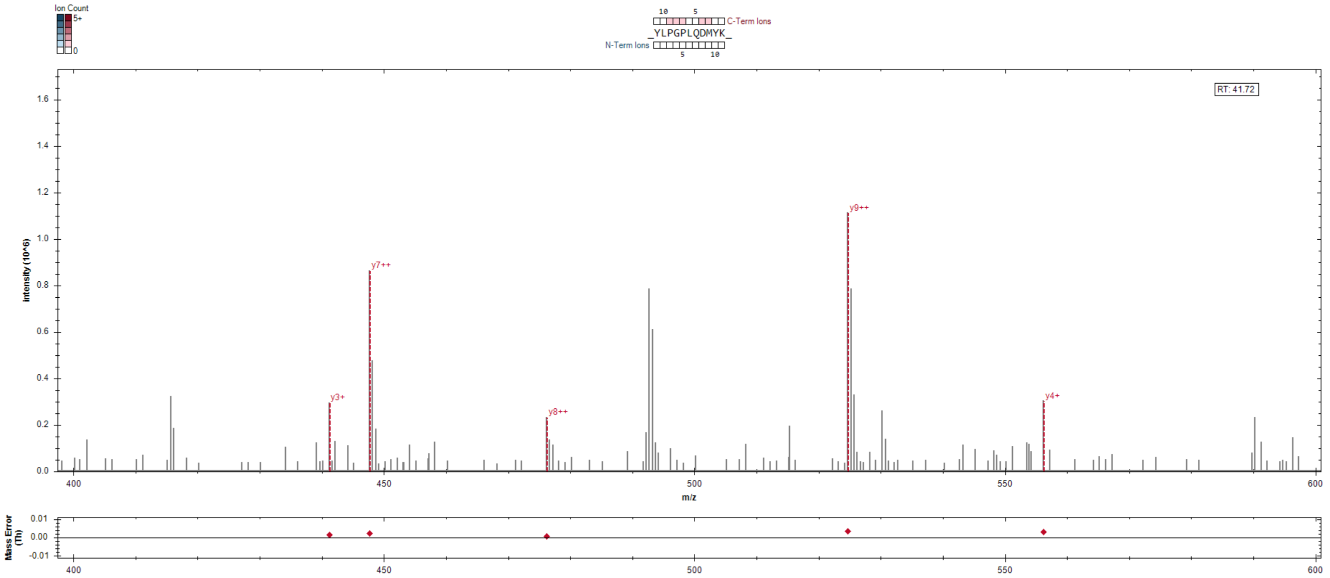
Ion Count
5+
0
C-Term Ions
YLPGPLQDMYK
N-Term Ions
RT: 41.72
Intensity (10^6)
y9++
y7++
y3+
y8++
y4+
m/z
Mass Error (Th)

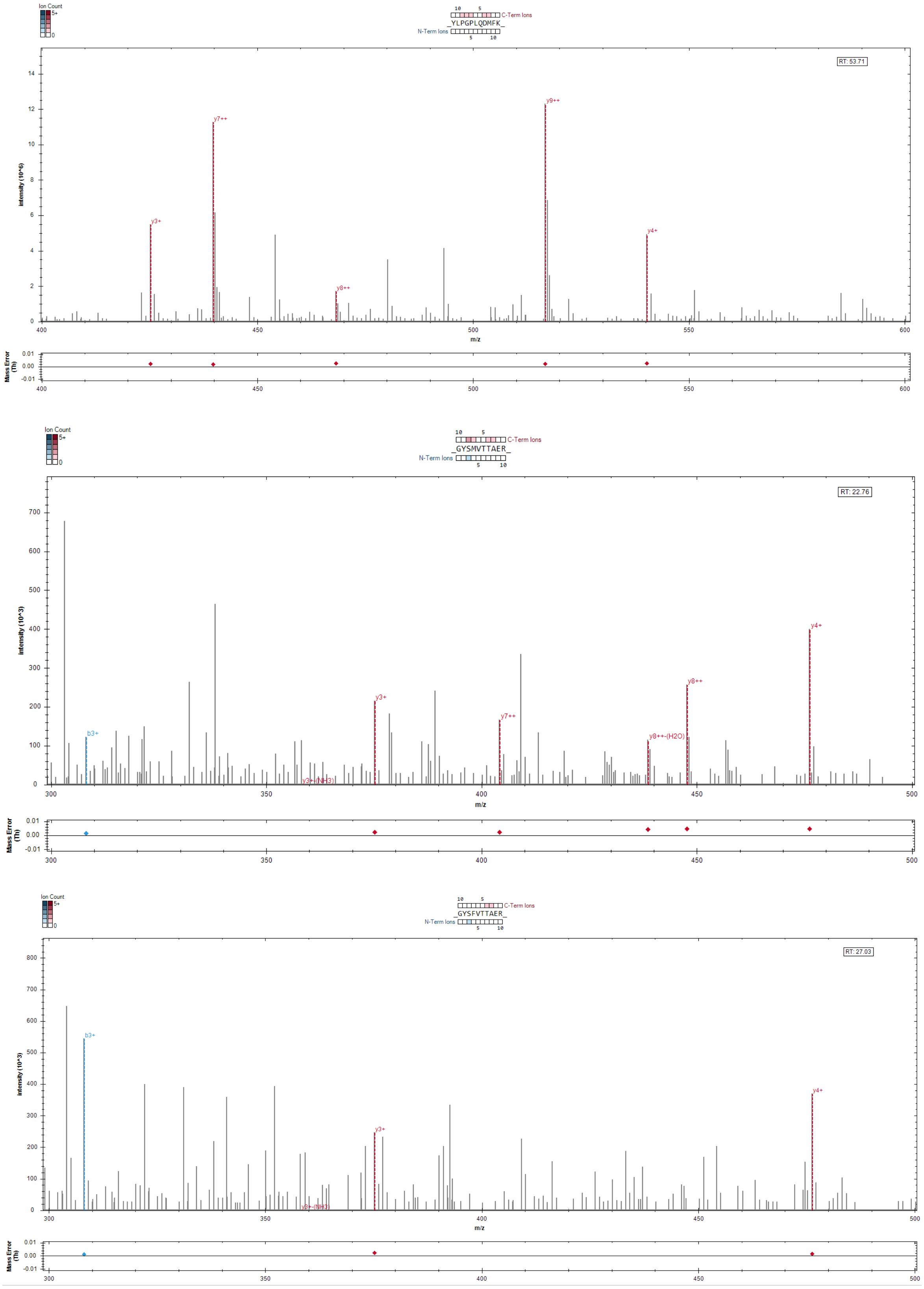

Supplementary Figure 14 related to Figure 6. Proteomic spectra of mistranslated peptides identified in the brains of *tRNA-Phe-1-1*$^{-/-}$ mice.

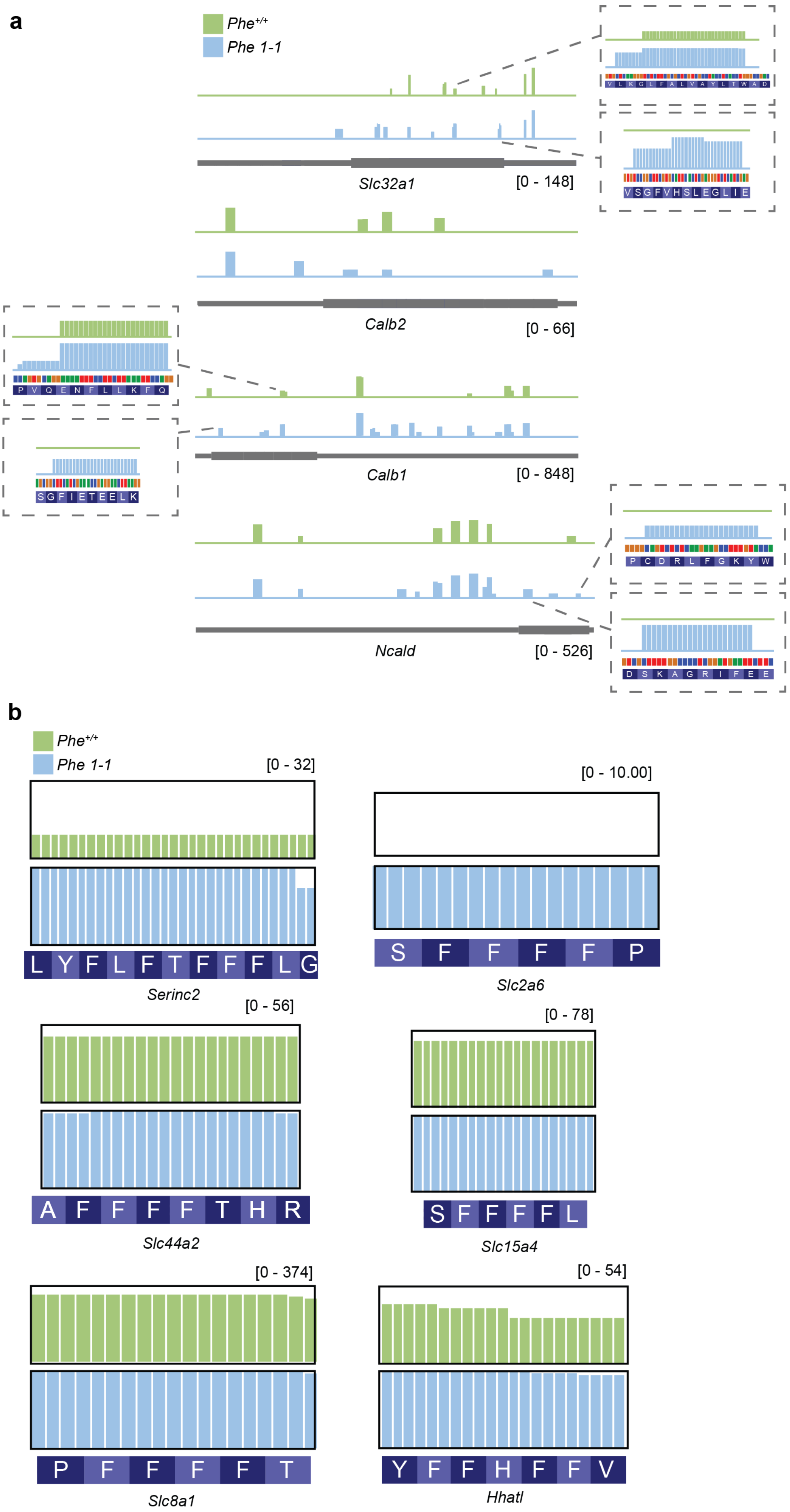

a
Phe+/+
Phe 1-1
Slc32a1
[0 - 148]
Calb2
[0 - 66]
Calb1
[0 - 848]
Ncald
[0 - 526]
b
Phe+/+
Phe 1-1
[0 - 32]
L Y F L F T F F F L G
Serinc2
[0 - 10.00]
S F F F F P
Slc2a6
[0 - 56]
A F F F F T H R
Slc44a2
[0 - 78]
S F F F F L
Slc15a4
[0 - 374]
P F F F F T
Slc8a1
[0 - 54]
Y F F H F F V
Hhatl

Supplementary Figure 15 related to Figure 6. Ribosome profiling of three control and three *tRNA-Phe-1-1*$^{-/-}$ mice identified stalling at phenylalanine codons in the brains of *tRNA-Phe-1-1* knockout mice. Cumulative reads per genotype of each stalled mRNA regions are shown in the square brackets. (**a**) Specific stalling was identified at phenylalanine codons in mRNAs encoding solute carriers involved in calcium-medicated neurotransmission. The stalled regions in the mRNAs from the *Phe-1-1* knockout mice are shown in the insets in blue and these stalled regions are reduced or absent in the control mice and are shown in green. (**b**) Examples of specific stalled regions within mRNAs with consecutive phenylalanine codons are shown.

Supplementary Methods

ATAC-Seq analysis

Sequenced reads were trimmed with Trim Galore[1] (0.6.4_dev) using cutadapt[2] (1.18) (with parameters: --paired --fastqc, corresponding to cutadapt parameters: -e 0.1 -q 20 -O 1 -a CTGTCTCTTATA). Trimmed reads were aligned to the mouse genome downloaded from GENCODE (GRCm38.p6, primary assembly, (ftp://ftp.ebi.ac.uk/pub/databases/gencode/Gencode_mouse/release_M24/GRCm38.primary_assembly.genome.fa.gz) on 21 March, 2018.) with Bowtie2[3] (2.4.1) (--very-sensitive --dovetail --no-discordant --no-mixed -k 20 -X 1000 --fr). PCR duplicates in the resulting BAM files were marked and removed with Picard Tools (http://broadinstitute.github.io/picard/; Broad Institute) (2.23.8) MarkDuplicates and filtered with SAMtools (Li et al., 2009) (1.10) (-q 30 -f 2 -F 256 -F 4), retaining only properly-paired primary alignments, removing low-quality alignments and those that fall within blacklisted regions (comprised of the ENCODE ENCFF547MET.bed blacklist[4] and the mitochondrial chromosome). Fragment size periodicity was estimated with the fast Fourier transform algorithm in the 01_calFragDist function of ATACgraph[5] and used to segment the data into nucleosome-free, mono-, di- and tri-nucleosome bound sets (based on a 175 nt periodicity). Nucleosome-free reads were subset with deeptools[6] (3.5.0) alignmentSieve (--maxFragmentLength 175) and peak calling was performed with Genrich (https://github.com/jsh58/Genrich) (0.6) (-E ENCFF547MET.bed -a 200 -m 30 -q 0.05 -j -r -e chrM,chrY -v) for each sample group. Generation of a consensus peakset and differential accessibility analysis of nucleosome-free regions was performed with DiffBind[7] (3.0.8) in R (https://www.R-project.org) (4.0.3), applying the internal mouse genome blacklist and counting reads with the parameters: summits=FALSE, mapQCth=30, fragmentSize=0. The native DESeq2 method was used for testing changes in chromatin accessibility between the knockouts and wild types (no greylist applied). Significant peaks were annotated with bedtools

intersect[8] and annotatePeaks.pl from the HOMER suite (4.11.1) against the mm10 (Dec 2011) gtRNAdb[9] tRNA and GENCODE vM24 (ftp://ftp.ebi.ac.uk/pub/databases/gencode/Gencode_mouse/release_M24/gencode.vM24. primary_assembly.annotation.gtf.gz on 20 April, 2020) gene annotations. Coverage profiles for each data subset were generated with deeptools bamCoverage (--extendReads --minFragmentLength [minlength] --maxFragmentLength [maxlength] --normalizeUsing CPM -bs 1 -of bigwig), after using the ATACshift function of deeptools alignmentSieve (--ATACshift) on the input BAMs, summarized with deeptools multiBigWigSummary (bins --outRawCounts -bs 1) and averaged per sample group. In addition to the genome-wide analysis, we also analysed tRNA regions specifically (using the gtRNAdb high-confidence set +/- 50 nt flanking regions), with the nucleosome-free BAMs using featureCounts[10] (2.0.0) (-p -B -C -P -d 20 -D 1000 -Q 30 -f -t 'exon' -g 'transcript_id') to count reads mapping to these regions and DESeq2[11] to test for changes, using apeglm[12] for effect size shrinkage[13], returning s-values.

Supplementary References:

1. Krueger, F. Trim Galore: A wrapper tool around Cutadapt and FastQC. (2015).

2. Martin, M. Cutadapt removes adapter sequences from high-throughput sequencing reads. *EMBnet journal* 17, 10–12 (2011).

3. Langmead, B. & Salzberg, S. L. Fast gapped-read alignment with Bowtie 2. *Nature Methods* 9, 357–359 (2012).

4. Amemiya, H. M., Kundaje, A. & Boyle, A. P. The ENCODE Blacklist: Identification of Problematic Regions of the Genome. *Sci Rep-uk* 9, 9354 (2019).

5. Lu, R. J.-H. *et al.* ATACgraph: Profiling Genome-Wide Chromatin Accessibility From ATAC-seq. *Frontiers Genetics* 11, 618478 (2021).

6. Ramírez, F. *et al.* deepTools2: a next generation web server for deep-sequencing data analysis. *Nucleic Acids Res* 44, W160–W165 (2016).

7. Stark, R. Research in Computational Molecular Biology, 16th Annual International Conference, RECOMB 2012, Barcelona, Spain, April 21-24, 2012. Proceedings. 286–286 (2012) doi:10.1007/978-3-642-29627-7_30.

8. Quinlan, A. R. & Hall, I. M. BEDTools: a flexible suite of utilities for comparing genomic features. *Nature Communications* 26, 841–842 (2010).

9. Chan, P. P. & Lowe, T. M. GtRNAdb 2.0: an expanded database of transfer RNA genes identified in complete and draft genomes. *Nucleic Acids Res* 44, D184–D189 (2016).

10. Liao, Y., Smyth, G. K. & Shi, W. featureCounts: an efficient general purpose program for assigning sequence reads to genomic features. *Bioinformatics* 30, 923–930 (2014).

11. Love, M. I., Huber, W. & Anders, S. Moderated estimation of fold change and dispersion for RNA-seq data with DESeq2. *Genome Biol* 15, 550 (2014).

12. Zhu, A., Ibrahim, J. G. & Love, M. I. Heavy-tailed prior distributions for sequence count data: removing the noise and preserving large differences. *Bioinformatics* 35, 2084–2092 (2019).

13. Stephens, M. False discovery rates: a new deal. *Biostat Oxf Engl* 18, 275–294 (2017).
