# Supplementary material for: Copy number variation in tRNA isodecoder genes impairs mammalian development and balanced translation
Source: Nat Commun. 2023 Apr 18;14:2210. doi: 10.1038/s41467-023-37843-9 (PMC10113395; doi:10.1038/s41467-023-37843-9)
Supplement: Supplementary file 3 — Description of Additional Supplementary Files [file 41467_2023_37843_MOESM3_ESM.pdf]

Description of Additional Supplementary Files

File Name: Supplementary Data 1
Description: Off target effects in WGS data

File Name: Supplementary Data 2
Description: Differential expression of genes in tRNA-Phe knockout mice compared to controls

File Name: Supplementary Data 3
Description: Protein changes in tRNA-Phe knockout mice compared to controls

File Name: Supplementary Data 4
Description: Northern blotting probe sequences
