# Supplementary material for: Copy number variation in tRNA isodecoder genes impairs mammalian development and balanced translation
Source: Nat Commun. 2023 Apr 18;14:2210. doi: 10.1038/s41467-023-37843-9 (PMC10113395; doi:10.1038/s41467-023-37843-9)
Supplement: Supplementary file 5 — Reporting Summary [file 41467_2023_37843_MOESM5_ESM.pdf]

nature portfolio

Corresponding author(s): Aleksandra Filipovska

Last updated by author(s): Mar 7, 2023

Reporting Summary

Nature Portfolio wishes to improve the reproducibility of the work that we publish. This form provides structure for consistency and transparency in reporting. For further information on Nature Portfolio policies, see our Editorial Policies and the Editorial Policy Checklist.

Statistics

For all statistical analyses, confirm that the following items are present in the figure legend, table legend, main text, or Methods section.

n/a	Confirmed	
☐	☒	The exact sample size (*n*) for each experimental group/condition, given as a discrete number and unit of measurement
☐	☒	A statement on whether measurements were taken from distinct samples or whether the same sample was measured repeatedly
☐	☒	The statistical test(s) used AND whether they are one- or two-sided<br>*Only common tests should be described solely by name; describe more complex techniques in the Methods section.*
☒	☐	A description of all covariates tested
☒	☐	A description of any assumptions or corrections, such as tests of normality and adjustment for multiple comparisons
☐	☒	A full description of the statistical parameters including central tendency (e.g. means) or other basic estimates (e.g. regression coefficient) AND variation (e.g. standard deviation) or associated estimates of uncertainty (e.g. confidence intervals)
☒	☐	For null hypothesis testing, the test statistic (e.g. *F*, *t*, *r*) with confidence intervals, effect sizes, degrees of freedom and *P* value noted<br>*Give P values as exact values whenever suitable.*
☒	☐	For Bayesian analysis, information on the choice of priors and Markov chain Monte Carlo settings
☒	☐	For hierarchical and complex designs, identification of the appropriate level for tests and full reporting of outcomes
☒	☐	Estimates of effect sizes (e.g. Cohen's *d*, Pearson's *r*), indicating how they were calculated

Our web collection on statistics for biologists contains articles on many of the points above.

Software and code

Policy information about availability of computer code

Data collection	Illumina DRAGEN germline alignment and variant calling workflow (v3.9.3), Cas-OFFinder, Trim Galore (0.6.4_dev), cutadapt, STAR, Salmon, DeepLabCut, Spectronaut version 14 and 15, fivepseq v1.0.0.
Data analysis	All software used is described in the methods section and code was deposited in GitHub as stated in the Data Availability Section. The website link is: https://github.com/ORAFLAB/Hughes_et_al_2023.

For manuscripts utilizing custom algorithms or software that are central to the research but not yet described in published literature, software must be made available to editors and reviewers. We strongly encourage code deposition in a community repository (e.g. GitHub). See the Nature Portfolio guidelines for submitting code & software for further information.

Data

Policy information about availability of data

All manuscripts must include a data availability statement. This statement should provide the following information, where applicable:

- Accession codes, unique identifiers, or web links for publicly available datasets
- A description of any restrictions on data availability
- For clinical datasets or third party data, please ensure that the statement adheres to our policy

Transcriptomic data are deposited to the Gene Expression Omnibus (GEO) as GSE223662, whole genome sequencing data are deposited in Biosamples as PRJNA777439 and the mass spectrometry proteomics data have been deposited to the ProteomeXchange (http://proteomecentral.proteomexchange.org)

PXD029684 via the PRIDE partner repository. ATAC-Seq data are deposited to the Gene Expression Omnibus (GEO) as GSE200783. The scripts for the analyses are deposited in GitHub: https://github.com/ORAFLAB/Hughes_et_al_2023. All of these data are now released and publicly available.

Human research participants

Policy information about studies involving human research participants and Sex and Gender in Research.

Reporting on sex and gender	n/a
Population characteristics	n/a
Recruitment	n/a
Ethics oversight	n/a

Note that full information on the approval of the study protocol must also be provided in the manuscript.

Field-specific reporting

Please select the one below that is the best fit for your research. If you are not sure, read the appropriate sections before making your selection.

☒ Life sciences ☐ Behavioural & social sciences ☐ Ecological, evolutionary & environmental sciences

For a reference copy of the document with all sections, see nature.com/documents/nr-reporting-summary-flat.pdf

Life sciences study design

All studies must disclose on these points even when the disclosure is negative.

Sample size	No statistical methods were used to predetermine sample size. The minimal sample size was n=3 determined in consultation with a biostatistician to achive statistically valid endpoints.
Data exclusions	No data were excluded from the analyses.
Replication	All experiments were performed with a minimum of three times independent replicates and replication was successful.
Randomization	No randomization was performed in this study, as no experimental groups were allocated.
Blinding	No blinding was performed in this study, as no group allocation was conducted.

Reporting for specific materials, systems and methods

We require information from authors about some types of materials, experimental systems and methods used in many studies. Here, indicate whether each material, system or method listed is relevant to your study. If you are not sure if a list item applies to your research, read the appropriate section before selecting a response.

Materials & experimental systems

n/a	Involved in the study	
☐	☒	Antibodies
☒	☐	Eukaryotic cell lines
☒	☐	Palaeontology and archaeology
☐	☒	Animals and other organisms
☒	☐	Clinical data
☒	☐	Dual use research of concern

Methods

n/a	Involved in the study	
☒	☐	ChIP-seq
☒	☐	Flow cytometry
☒	☐	MRI-based neuroimaging

Antibodies

Antibodies used	Calretinin (ab92341) Abcam, diluted 1:500. Rabbit polyclonal antibody: Calbidin-D-28K (C2724) Sigma, diluted 1:500. Mouse monoclonal antibody: beta Actin (ab6276) Abcam, diluted 1:1000. IRDye 800CW goat anti-rabbit immunoglobulin G (IgG) or IRDye 680LT goat anti-mouse IgG (LI-COR Biosciences) and donkey anti-rabbit Alexa Fluor 555 (A-31572, Thermo Fisher) secondary antibodies were used.

Validation	All primary antibodies were commercially obtained from Abcam and their validation is available on the Abcam website (https://www.abcam.com). The antibodies were validated by histological staining or western blotting of human, mouse or rat tissues and lysates, respectively.

Animals and other research organisms

Policy information about studies involving animals; ARRIVE guidelines recommended for reporting animal research, and Sex and Gender in Research

Laboratory animals	We used mice (Mus musculus) on a C57BL/6N background strain that were 10 weeks old.
Wild animals	The study did not involve wild animals.
Reporting on sex	The results in the study are based on both genders and the gender of the mice was assigned based on visual inspection. We have indicated throughout the methods the genders used for our analyses.
Field-collected samples	The study did not involve samples collected from the field.
Ethics oversight	The Animal Ethics Committee of The University of Western Australia approved the work.

Note that full information on the approval of the study protocol must also be provided in the manuscript.
