# Supplementary material for: Copy number variation in tRNA isodecoder genes impairs mammalian development and balanced translation
Source: Nat Commun. 2023 Apr 18;14:2210. doi: 10.1038/s41467-023-37843-9 (PMC10113395; doi:10.1038/s41467-023-37843-9)

Figure 2b source data

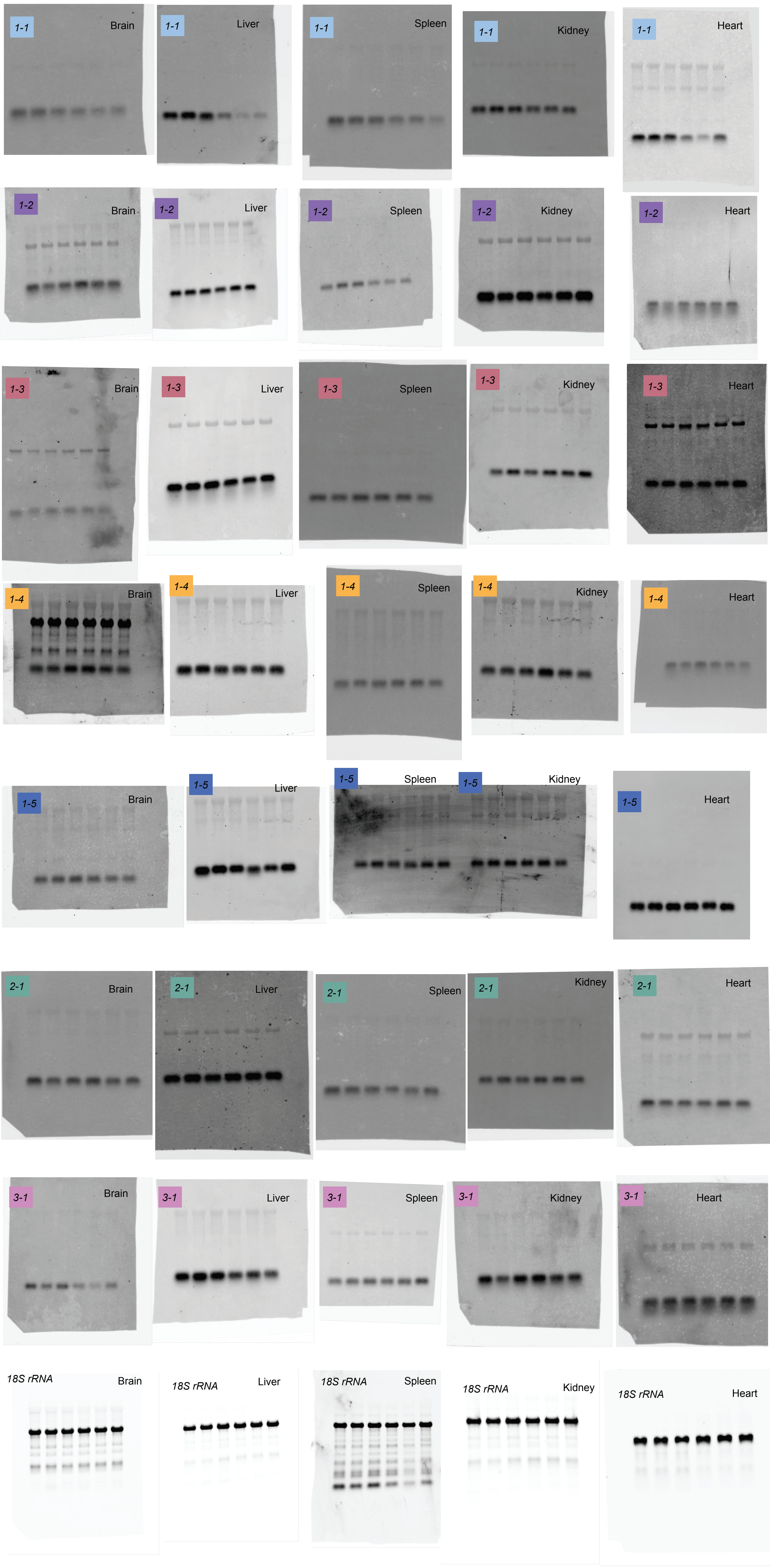

Figure 5c source data

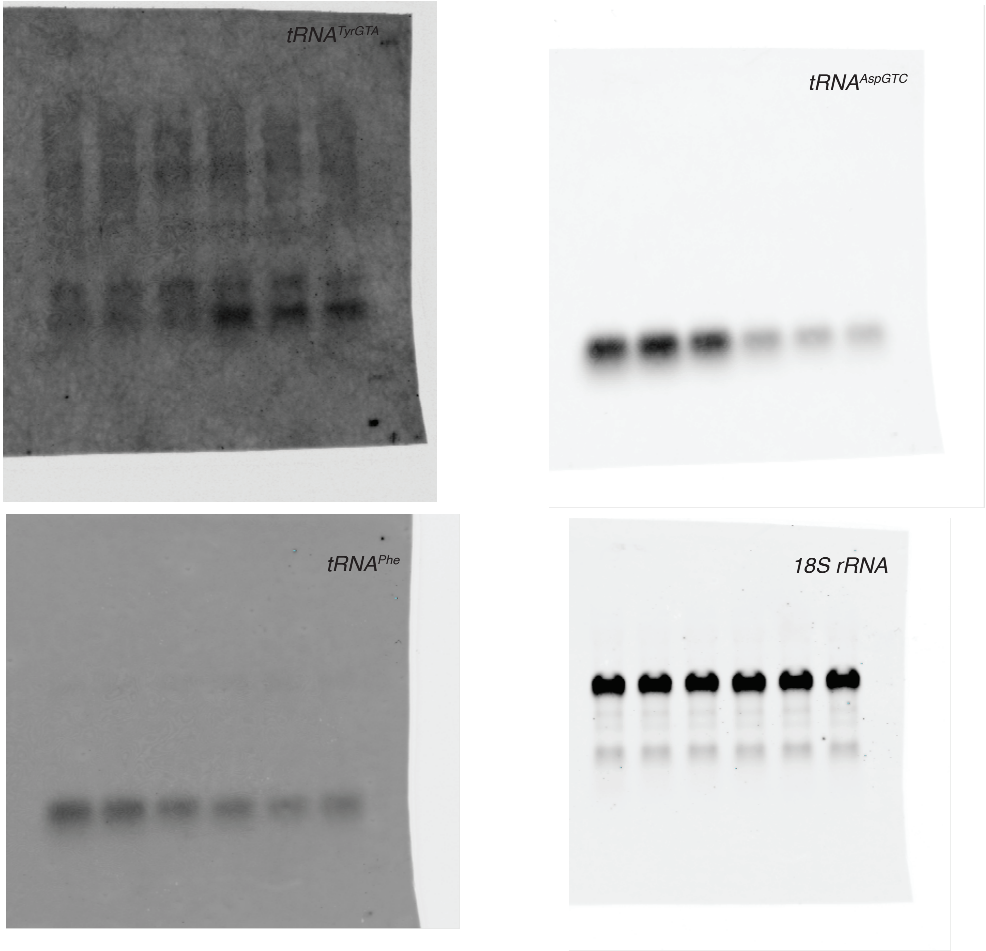

Figure 6e source data

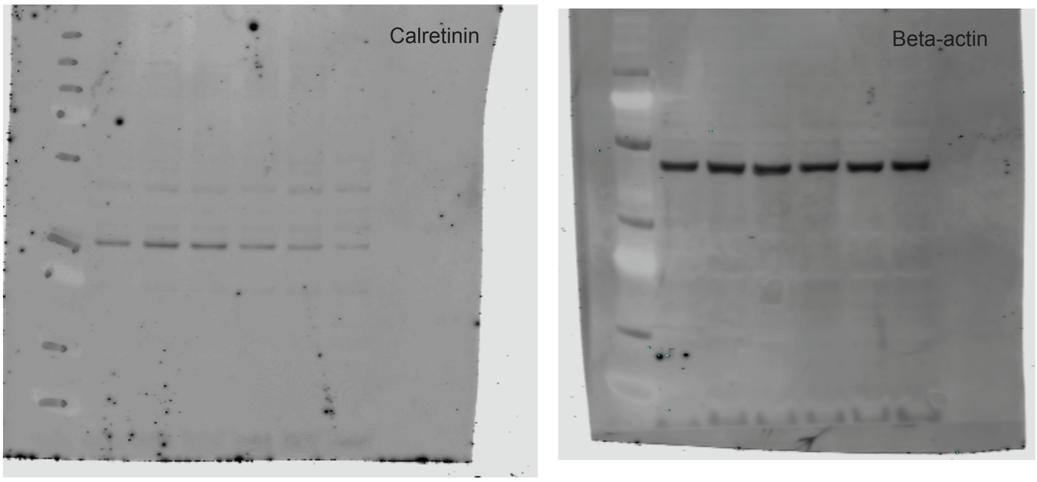

Supplementary Figure 11c source data

tRNALeuCAA Liver

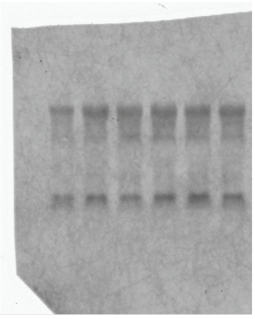

tRNATyrGTA Liver

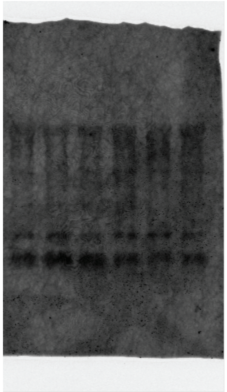

tRNAAspGTC Liver

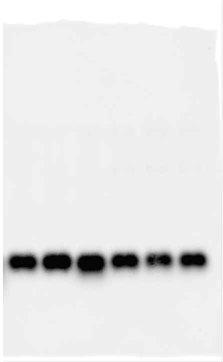

tRNAPhe Liver

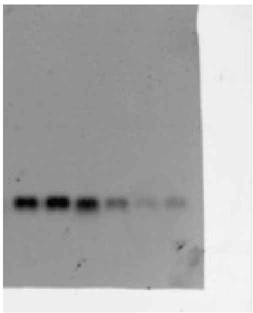

18S rRNA Liver

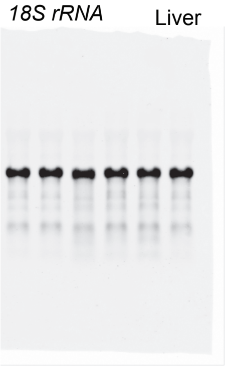

Supplement: Supplementary file 6 — Source Data [file 41467_2023_37843_MOESM6_ESM.pdf]
